# Turning universal O into rare Bombay type blood

**DOI:** 10.1038/s41467-023-37324-z

**Published:** 2023-03-30

**Authors:** Itxaso Anso, Andreas Naegeli, Javier O. Cifuente, Ane Orrantia, Erica Andersson, Olatz Zenarruzabeitia, Alicia Moraleda-Montoya, Mikel García-Alija, Francisco Corzana, Rafael A. Del Orbe, Francisco Borrego, Beatriz Trastoy, Jonathan Sjögren, Marcelo E. Guerin

**Affiliations:** 1 Structural Glycobiology Laboratory, Biocruces Bizkaia Health Research Institute, Cruces University Hospital, 48903 Barakaldo, Bizkaia Spain; 2 Structural Glycobiology Laboratory, Center for Cooperative Research in Biosciences (CIC bioGUNE), Basque Research and Technology Alliance (BRTA), Bizkaia Technology Park, Building 801A, 48160 Derio, Spain; 3 Genovis AB, Box 790, 22007 Lund, Sweden; 4 Immunopathology Group, Biocruces Bizkaia Health Research Institute, Cruces University Hospital, 48903 Barakaldo, Bizkaia Spain; 5 Departamento Química and Centro de Investigación en Síntesis Química, Universidad de La Rioja, 26006 Logroño, Spain; 6 Hematology and Hemotherapy Service, Cruces University Hospital, Biocruces Bizkaia Health Research Institute, 48903 Barakaldo, Bizkaia Spain; 7 Ikerbasque, Basque Foundation for Science, 48009 Bilbao, Spain

**Keywords:** Glycobiology, Enzymes, X-ray crystallography, Bacteria

## Abstract

Red blood cell antigens play critical roles in blood transfusion since donor incompatibilities can be lethal. Recipients with the rare total deficiency in H antigen, the O_h_ Bombay phenotype, can only be transfused with group O_h_ blood to avoid serious transfusion reactions. We discover FucOB from the mucin-degrading bacteria *Akkermansia muciniphila* as an α-1,2-fucosidase able to hydrolyze Type I, Type II, Type III and Type V H antigens to obtain the afucosylated Bombay phenotype in vitro. X-ray crystal structures of FucOB show a three-domain architecture, including a GH95 glycoside hydrolase. The structural data together with site-directed mutagenesis, enzymatic activity and computational methods provide molecular insights into substrate specificity and catalysis. Furthermore, using agglutination tests and flow cytometry-based techniques, we demonstrate the ability of FucOB to convert universal O type into rare Bombay type blood, providing exciting possibilities to facilitate transfusion in recipients/patients with Bombay phenotype.

## Abstract (web summary)

People with the rare Bombay-type O_h_ blood group can only be transfused with O_h_ blood. Here, the authors characterize a bacterial α−1,2-fucosidase that can convert universal O type into rare Bombay type blood.

## Introduction

Blood group antigens play fundamental roles not only in blood transfusion but also in organ transplantation. Blood group antigens are assigned to blood group systems based on their relationship to each other as determined by serological or genetic criteria^1,2^. They are based either on oligosaccharide epitopes, including the ABO, P, and Lewis antigens, or specific amino acid sequences, such as Rh, Kell, and Duffy antigens^3^. There are currently 43 recognized blood group systems containing 345 red cell antigens. The 43 systems are genetically determined by 48 genes (https://www.isbtweb.org/). The blood group antigens are not restricted solely to red blood cells (RBCs) or even to hematopoietic tissues. They are also widely distributed throughout the human body such as the salivary glands, gastrointestinal and urinary tracts, and respiratory cavities^4^. Up to date, and besides their importance, the physiological functions of several blood group antigens are still unknown.

**Fig. 1:**
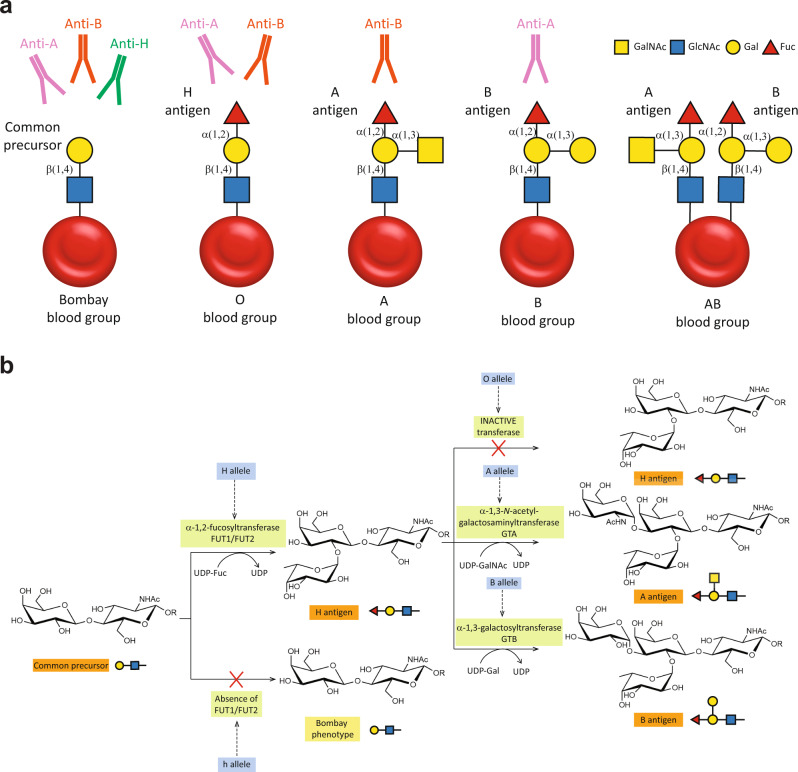
A, B, H, and Bombay antigens in RBCs. **a** Cartoon representation of Bombay, O, A, B, and AB blood groups antigen’s carbohydrate epitopes present in RBC surface (RBC are depicted as red circles) and antibodies. **b** Enzymatic pathway for the biosynthesis of A, B, and H antigens. Enzymes involved in biosynthesis are highlighted in light green, encoding genes in blue and antigen’s carbohydrate epitopes in orange and yellow.

The most well-known and clinically relevant blood groups are ABO. Discovered in 1900 by Karl Landsteiner through agglutination tests, the antigens present in these groups are composed of specific oligosaccharides mainly linked to proteins (ca. 90%) and, to a lesser extent, to lipids (ca. 10%)^5–7^. The antigens are classified as A, B, or H and subclassified depending on the sugar composition and the variety of linkages^8^ (Fig. 1a). The A, B, and H antigens are formed by the sequential action of glycosyltransferases encoded by three genetic loci, the *ABO*, *H*, and *Secretor* [Se], now termed the *ABO*, *FUT1*, and *FUT2* loci^9^. Blood group antigen synthesis is initiated by the addition of a fucose residue to a common precursor glycan chain (six different types are known) to obtain the H antigen^10,11^. Two fucosyltransferases are involved in this reaction. The *H* allele encodes an α-1,2-fucosyltransferase (FUT1) that transfers a fucose residue to Type II and Type IV glycan units to form the H antigen on erythrocytes and vascular endothelial cells^9^. The *Se* allele encodes another α-1,2-fucosyltransferase (FUT2) that uses Type I and Type III precursors as acceptors to form the H antigen in the epithelia of the gastrointestinal, respiratory, and reproductive tracts, and salivary glands, as well as modifying milk oligosaccharides to generate the H antigen^9^. A and B antigens are subsequently synthesized from the H antigen by specific glycosyltransferases encoded by the *A*, *B*, or *O* alleles of the *ABO* locus (chromosome 9q34.1-q34.2)^10,12,13^. The *A* allele encodes an α-1,3-acetylgalactosaminyltransferase (GTA) which transfers an *N*-acetylgalactosamine residue to a galactose residue present in the H antigen to obtain the A antigen. The *B* allele encodes an α-1,3-galactosaminyltransferase (GTB), which transfers a galactose residue to the third position of the galactose residue present in the H antigen to obtain the B antigen^12–14^. In contrast, *O* alleles at the ABO locus encode a functionally inactive A/B glycosyltransferase (Fig. 1b). Therefore, depending on the genetic heritage, 4 blood groups can be defined: (i) blood group A if antigen A is present, (ii) blood group B if antigen B is present, (iii) blood group AB group if both A and B antigens are present (A and B alleles could be both co-expressed) or (iv) blood group O if only antigen H is present^15^. Importantly, individuals presenting a specific antigen (or antigens) lack antibodies directed against it (them) while having antibodies for the antigens they do not express. Therefore, A and B blood group individuals present B (anti-B) or A (anti-A) antibodies, respectively. AB blood group individuals present neither anti-A nor anti-B antibodies, whereas H blood group individuals present both (Fig. 1a)^8^. These antibodies are responsible for acute intravascular transfusion reactions and acute transplant rejections due to incompatible blood or organ^8^. In addition, they can cause hemolytic disease of the newborn (HDN)^16^, a major cause of fetal loss and death among newborn babies. HDN due to ABO incompatibility is usually less severe than Rh incompatibility. Supporting this notion, (i) fetal RBCs express less of the ABO blood group antigens compared with adult levels and (ii) the ABO blood group antigens are expressed by a variety of fetal (and adult) tissues, reducing the chances of anti-A and anti-B binding their target antigens on the fetal RBCs^16^.

In 1952 a new rare blood group related to the ABO system was first described in the city of Bombay (currently Mumbai), India^17,18^. The serum samples showed the presence of anti-A, anti-B, and anti-H antibodies capable of agglutinating RBCs of the A, B, AB, and O groups. A close inspection into the genome sequences revealed that mutations in the two homozygous recessive alleles, “*h/h*” of *FUT1* and “*se/se*” of *FUT2* genes lead to no expression of any of the α-1,2-fucosyltransferases^15,19^. The consequence is a phenotype called Bombay or O_h_ phenotype. Para-Bombay or H+^W^ phenotype can also be distinguished when a low amount of H antigen is expressed in two different situations: (i) when the lack of H antigen is caused by the inactive of the *FUT1* gene product but the *FUT2* gene product is still active (therefore, H antigen is found in secretions, Se) or (ii) when a mutation in the *FUT1* gene leads to a less active fucosyltransferase in combination with active or inactive *FUT2* gene product (Se or se, respectively)^15^.

It is considered a rare blood group because it affects <1 person per 2000 of the general population and shows a geographically asymmetric distribution. Lower frequency is found in the European population (1:1,000,000)^15^ compared to the prevalence in Iran (1:125,000)^20^ or Mumbai, India (1:10,000)^11^. Moreover, the Southern and Western regions of India show the greatest number of Bombay phenotype populations, with the highest frequency in Bhuyan tribal population in Orissa, revealing the average prevalence of the Bombay phenotype to be 1 in 278^21^. Endogamy and consanguinity might be the main causes of the high prevalence of the rare Bombay blood group in India because they facilitate the homozygous expression of its rare recessive genetic character^20,22^. The presence of anti-H antibodies in Bombay and para-Bombay phenotypes, mostly IgM natural antibodies, can cause severe hemolytic transfusion reactions with intravascular hemolysis if the blood is combined with any other ABO blood group samples. This is why individuals with this characteristic phenotype need to be transfused with the same blood group samples^15^.

In this work, we discover Amuc_1120 from the mucin-degrading bacteria *A. muciniphila* as an α-1,2-fucosidase able to hydrolyze all Types of H antigen to obtain the afucosylated Bombay phenotype. We decided to name Amuc_1120 as FucOB, α-1,2-L-Fucosidase O to Bombay hereafter. We provide high-resolution X-ray crystal structures of FucOB, which show a three-domain architecture, including a GH95 glycoside hydrolase (GH). In combination with thorough structural/biochemical comparisons with other GH95 family members, in silico molecular docking calculations, molecular dynamics simulations, extensive site-directed mutagenesis, and enzymatic activity methods we unravel the molecular basis of FucOB catalytic and substrate recognition mechanisms. Furthermore, using two complementary agglutination test assays and flow cytometry-based techniques, we demonstrate the ability of FucOB to convert O-type blood into rare Bombay-type blood. We propose that FucOB could be used as a biotechnological and therapeutic tool to facilitate blood transfusion in patients with Bombay phenotype.

## Results

### FucOB is an α-1,2-fucosidase that specifically cleaves H antigen

*A. muciniphila* is an anaerobic Gram-negative bacterium from the phylum Verrucomicrobia that promotes a beneficial effect on human health, likely based on the regulation of mucus thickness and gut barrier integrity, but also the modulation of the immune system^23,24^. *A. muciniphila* hydrolyzes up to 85% of the chemical structures of mucin orchestrated by different enzymes, mainly in the form of proteases, sulfatases, and GHs^25–27^. We have recently unveiled the molecular mechanism of *O*-glycan recognition and specificity for OgpA from *A. muciniphila*, a paradigmatic *O*-glycopeptidase that exclusively hydrolyzes the peptide bond N-terminal to serine or threonine residues substituted with an *O*-glycan^28,29^. The careful inspection of the protein-encoding genes in the genome of *A. muciniphila* ATCC BAA-835 strain showed that *ogpA* (*Amuc_1119*) is close to (i) a putative GH of the GH95 family (*Amuc_1120* or *fucOB*) and (ii) a predicted sulfatase (*Amuc_1118*; Supplementary Fig. 1). In particular, the GH95 family comprises 4313 amino acid sequences, of which only 17 enzymes have been biochemically characterized (Supplementary Table 1), including three reported activities: α-L-fucosidase (EC 3.2.1.51), α-1,2-L-fucosidase (EC 3.2.1.63), and α-L-galactosidase activities (EC 3.2.1.-)^30,31^. The expression of FucOB was found to be significantly upregulated under mucin versus glucose conditions suggesting that the enzyme could cooperate in the specific degradation of mucins^29,32^.

**Fig. 2:**
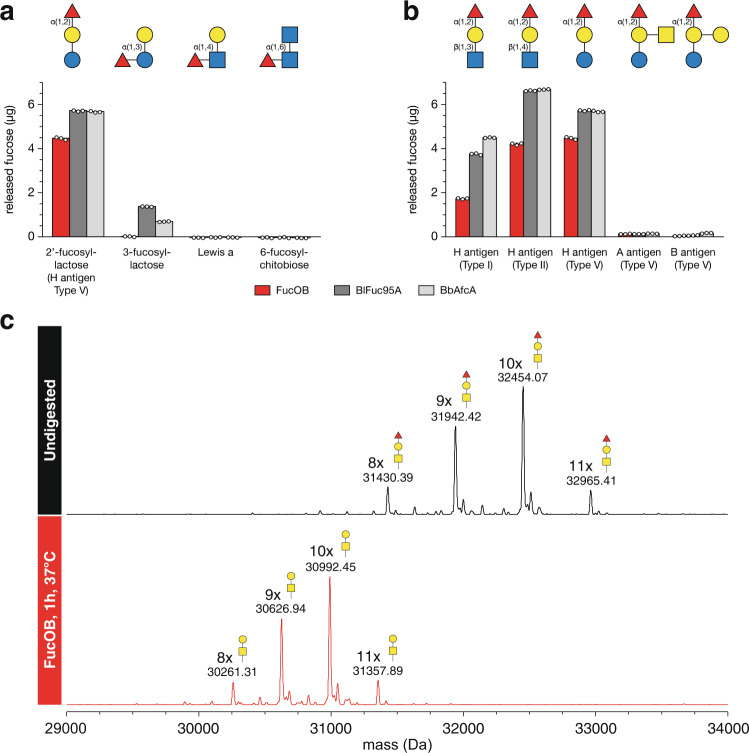
Substrate specificity of FucOB. **a**, **b** Quantification of released fucose after incubation of synthetic oligosaccharides with FucOB, *Bi*Fuc95A, and *Bb*AfcA for 30 min at 37 °C. Free fucose levels were quantified spectrophotometrically and compared to a standard curve of known concentrations. Bars and error bars represent the mean and standard deviation of triplicate measurements (shown as three empty dots). **c** Deconvoluted mass spectra of the TNFR fragment of glycoengineered etanercept before (black) and after (red) incubation with FucOB for 1 h at 37 °C. The major peaks are annotated with average mass and glycan composition. Source data are provided as a Source Data file.

To study the enzymatic activity and substrate specificity of FucOB, we purified the enzyme to apparent homogeneity (Supplementary Fig. 2; see the “Methods” section for details). FucOB from *A. muciniphila* comprises 796 residues (UniProt code B2UR61; GeneBank code ACD04946.1) with a predicted signal peptide (residues 1–23) that was removed from the construct. We incubated FucOB with a series of fucosylated oligosaccharides and quantified the amount of released fucose in vitro. Although more than one GH activity has been reported for this family, FucOB was very specific to L-fucose, which is equivalent to 6-deoxy-L-galactose (Fig. 2a; Supplementary Table 1). FucOB displays α-1,2-L-fucosidase activity and showed no activity against α-1,3, α-1,4, and α-1,6 fucosylated oligosaccharides (Fig. 2a). It is worth noting that the Fucα1-2Galβ epitope is part of the oligosaccharide anchor of blood group A, B and H antigens, present in many human tissue surfaces such as RBC or gastrointestinal epithelium. We, therefore, evaluated the ability of FucOB to process the α-1,2 linked fucose residue on synthetic oligosaccharides representing Type I, Type II, Type V H antigens, Type V A antigens and Type V B antigens. Interestingly, we found that FucOB hydrolyzes all three types of H antigen structures to obtain the afucosylated Bombay phenotype and very poorly hydrolyze the α-1,2 linked fucose in the branched structures of the A or B antigens (Fig. 2b; see the “Methods” section). We further compared the enzymatic activity and substrate specificity of two FucOB homologs from family GH95, *Bb*AfcA from *Bifidobacterium bifidum* (29% identity with FucOB) and Fucosidase 95A from *Bifidobacterium longum* CZ0511 (*Bi*Fuc95A; 29% identity with FucOB; NZYTech). As depicted in Fig. 2a, b, both *Bb*AfcA and *Bi*Fuc95A are a bit faster than FucOB in cleaving α-1,2 fucose but clearly less specific with activity also towards α-1,3 fucose as well. These results highlight the value of FucOB, a very specific enzyme for α-1,2-L-fucosylated substrates, and shows no activity against α-1,3, α-1,4, and α-1,6 fucosylated substrates. Lastly, we assessed the ability of FucOB to hydrolyze fucose not just from synthetic oligosaccharides but also in the context of a larger glycoconjugate such as a glycoprotein. To this end, we glycoengineered the TNFα receptor to carry 8–11 α-1,2-fucosylated core 1 *O*-glycans, corresponding to Type III H antigen structures, and used this as a model substrate to assay FucOB activity. After 1 h of incubation, the substrate was completely defucosylated by FucOB as demonstrated by reverse phase LC-MS (Fig. 2c).

### The architecture of full-length FucOB

**Fig. 3:**
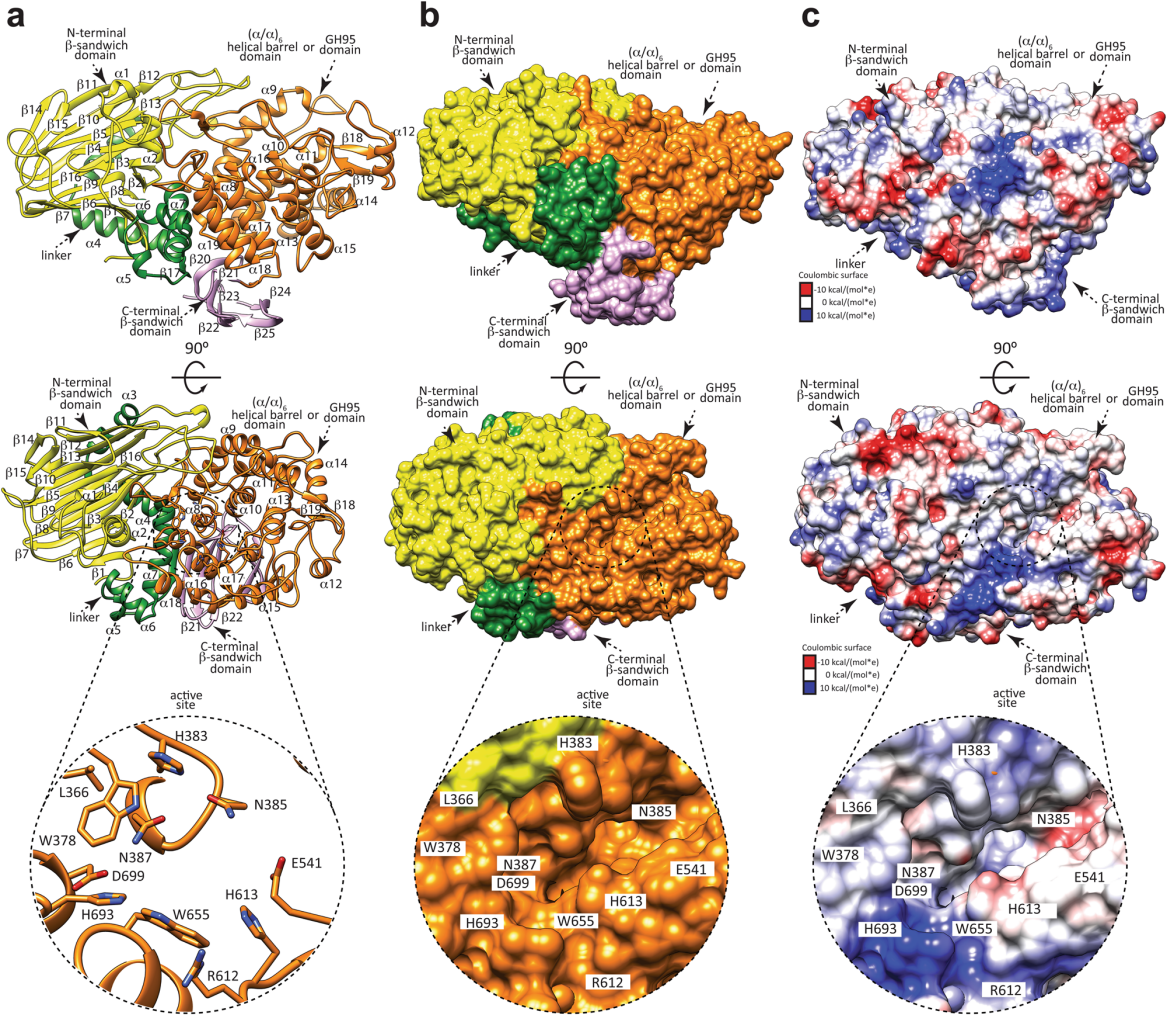
The overall structure of FucOB. **a** Two views of the cartoon representation showing the general fold and secondary structure organization of FucOB, including the catalytic (α/α)_6_ helical barrel domain or GH95 catalytic domain (orange) and the N- and C-terminal β-sandwich domains (yellow and pink, respectively). The bottom panel corresponds to a close-up view of the active site, shown as a cartoon/stick representation. **b** Two views of the surface representation of FucOB. The bottom panel represents a close-up view of the active site of FucOB showing the substrate binding pocket. **c** Two views of the electrostatic surface representation of FucOB showing the location of the putative substrate binding site and the catalytic site. The bottom panel shows a close-up view of the active site of FucOB showing the substrate binding pocket.

The crystal structure of the unliganded form of full-length FucOB was determined at 1.8 Å resolution using molecular replacement methods (FucOB; Supplementary Table 2; the structural model used for molecular replacement corresponds to the PDB code 2EAB; see the “Methods' section). The high quality of the electron density maps allowed the trace of residues 25–785 (Supplementary Fig. 3). A close inspection of the crystal structure revealed that FucOB crystallized as a monomer comprising three domains from the N- to the C-terminus: (i) a β-sandwich domain (residues 25–260), (ii) an (α/α)_6_ helical barrel catalytic domain (residues 357–712) and (iii) a second β-sandwich domain (residues 713–785; Fig. 3a–c). It is worth noting that the N-terminal β-sandwich domain is connected to the catalytic helical barrel domain through a linker comprised of five α-helices (α3, α4, α6, α7 and a short α5; residues 261–356; Fig. 3a–c). The first β-sheet of the N-terminal β-sandwich domain consists of nine β-strands with topology β1–β6–β7–β8–β9–β16–β13–β12–β11 (β6, β8, β16, β12 are antiparallel), whereas the second β-sheet comprises seven β-strands with topology β2–β3–β4–β5–β10–β15–β14 (β2–β4–β10–β14 are antiparallel), with an overall size of 41 Å × 40 Å × 11 Å (Fig. 3a–c). The central catalytic core (α/α)_6_ helical barrel domain consists of 12 α-helices (α8 to α19; Fig. 3a–c). The C-terminal β-sandwich domain consists of a β-sheet of five β-strands with topology β17–β20–β21–β22–β23 (β20–β22–β23 are antiparallel), whereas the second β-sheet comprises eight β-strands with topology β24–β25 (β25 is antiparallel), with an overall size of 27 Å × 17 Å × 10 Å.

The (α/α)_6_ helical barrel of FucOB displays a deep groove where the active site is located, mainly flanked by solvent-exposed and flexible loops, including loop 25 (α7−α8; residues 357–386), loop 28 (α9−α10; residues 429–458), loop 33 (β19–α13; residues 547–550), loop 35 (α14−α15; residues 587–614); loop 37 (α16−α17; residues 651-655), loop 39 (α18−α19; residues 683–699) and α-helix 8 (residues 387–392) (Fig. 3a–c). A first glycerol molecule is deeply buried into a pocket defined by W378, H383, W655, and H693. The O1 and O2 atoms make hydrogen bonds with the side chains of N387 and Q697, respectively. A second glycerol molecule accommodates into an adjacent solvent exposed pocket mainly defined by Y106 and W453. The O3 and O2 atoms make hydrogen bonds with the side chains of S444 and H383, respectively. The two glycerol molecules are involved in additional hydrogen-bonding interactions with a few water molecules. A third glycerol molecule is located near the first glycerol molecule. The O1, O2, and O3 atoms interact with the side chains of H693, R612, and H613, respectively. FucOB as well as other members of the GH95 family follow a single displacement inverting catalytic mechanism^30^. A conserved glutamic acid (E541 in FucOB; Supplementary Fig. 4) acts as a general acid catalyst. Mutation of E541 by alanine completely abolished the hydrolytic activity of the enzyme (see below). There is no carboxylic acid residue at the appropriate position for a general base catalyst, therefore, it is proposed that a water molecule acts as a general base in the reaction, activated by two asparagine residues (N385 and N387 in FucOB) and an aspartic acid residue (D699 in FucOB) to perform the nucleophilic attack to the fucose^30,33^ (Supplementary Fig. 4).

A search for structural homologs using the DALI server revealed that FucOB shows structural similarity to the four members of the GH95 family for which, to date, experimental structural data have been reported: (i) α-1,2-fucosidase *Xac*Afc95 from *Xanthomonas citri* (PDB code 7KMQ; *Z*-score of 42; root mean squared deviation (r.m.s.d.) value of 1.8 Å for 679 aligned residues; 35% identity)^34^, (ii) a putative GH95 member from *Bacillus halodurans* (PDB code 2RDY; *Z*-score of 41.3; r.m.s.d. value of 1.9 Å for 675 aligned residues, 36% identity), (iii) α-1,2-fucosidase *Bb*AfcA from *B. bifidum* (PDB code 2EAB; *Z*-score of 41.3; r.m.s.d. value of 2.2 Å for 698 aligned residues; 33% identity)^33^ and (iv) α-L-galactosidase BACOVA_03438 from *Bacteroides ovatus* (PDB code 4UFC; *Z*-score of 40.5; r.m.s.d. value of 1.8 Å for 677 aligned residues; 35% identity)^35^ (Supplementary Figs. 5-7). Interestingly, the genome of *A. muciniphila* strain ATCC BAA-835 encodes solely one additional enzyme that belongs to the GH95 family, Amuc_0186^36^, which shares 29% sequence identity with FucOB.

### Structural basis of H antigen recognition and specificity by FucOB

To further understand the FucOB substrate specificity at the molecular level, we performed co-crystallization experiments with (i) wild-type FucOB and (ii) the catalytically inactive variant FucOB_E541A_, both in the presence of V Type H blood group antigen as the substrate. Despite many efforts, we could not crystallize FucOB or FucOB_E541A_ in the presence of the substrate or the corresponding product. The structural comparison of FucOB and the FucOB_E541A_ revealed that the protein structure is mostly preserved and that there are no substantial conformational changes (r.m.s.d. of 0.477 Å for 761 residues). The crystal packing analysis of FucOB and FucOB_E541A_ structures reveals a strong π–π stacking interaction between W453 with two prolines residues, P765 and P783, of the neighbor protomer. Consequently, this protomer restricts the entrance of the substrate into the active site, supporting the inability to obtain a complex crystal form.

**Fig. 4:**
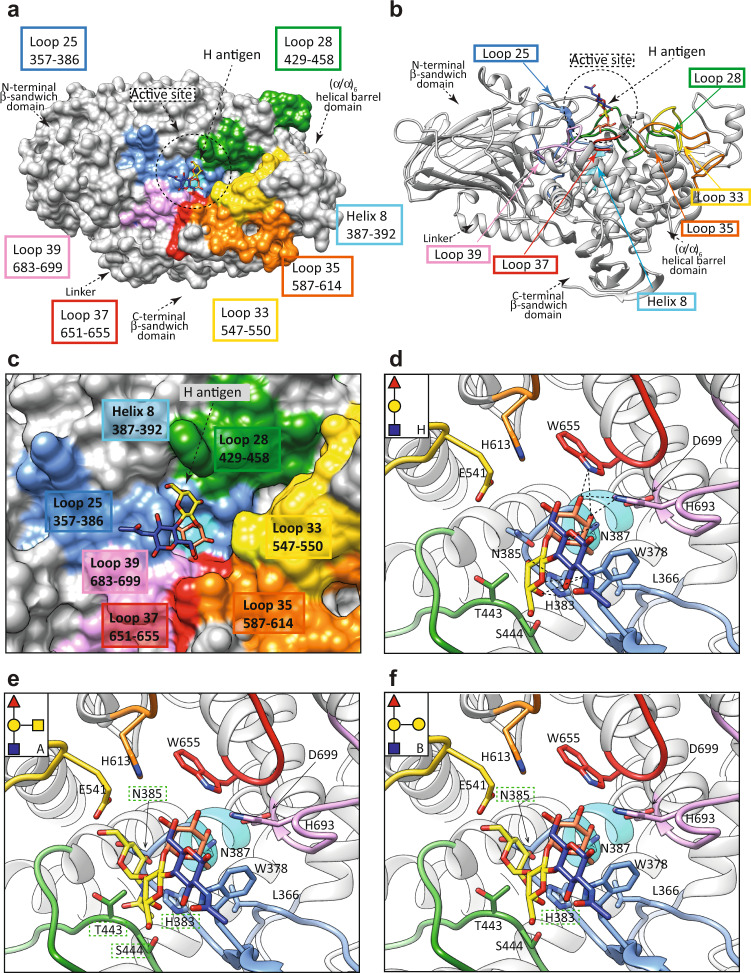
Structural basis of FucOB specificity for H-type blood antigen. **a** Surface representation of the FucOB structure, with annotated domains and loops, showing the location of the Type II H antigen substrate. **b** Cartoon representation of the FucOB structure, showing the location of the Type II H antigen substrate. **c** Surface representation of FucOB showing the location of the Type II H antigen into the active site. Docking calculations of FucOB with different blood group antigens including **d** Type II H, **e** Type II A, and **f** Type II B. The predicted clashes are shown as green dotted squares and H bonds are shown as black dotted lines.

To describe the architecture of the H-type blood group epitope binding site, we thus generated a three-dimensional model of FucOB in complex with H, A, and B antigens by in silico molecular docking calculations. To this end, we defined the putative FucOB substrate binding site considering the crystal structure of *Bb*AfcA in complex with the substrate 2’-fucosyllactose (2’FL; Fucα1-2Galβ1-4Glc; PDB code 2EAD). The H antigen’s O4 and O5 atoms of the galactose ring make hydrogen bonds with W378, whereas O3 and O4 atoms make hydrogen bonds with H383, both residues located in loop 25. In contrast, the fucose residue in H antigen is more deeply buried in the active site of FucOB. The O4 atom makes a hydrogen bond with H693 in loop 39; O5 makes a hydrogen bond with N387 located in α-helix 8; whereas O4 and O3 atoms make hydrogen bonds with W655 in loop 37 (Fig. 4a–d). Conversely, molecular docking calculations for the A-type blood group epitope containing the GalNAcα1-3(Fucα1-2)Gal oligosaccharide show that the GalNAc residue exhibits significant clashes with the protein (Fig. 4e). Specifically, GalNAc residue’s ring shows clashes, including C1 and C2 with T443 located in loop 28, and C2, C3, C4, O3 and O4 with N385 located in loop 25. Moreover, the acetyl group clashes with T443 and S444 in loop 28, and H383 in loop 25. Furthermore, molecular docking calculations for the B-type blood group epitope, containing the Galα1-3(Fucα1-2)Gal oligosaccharide, show similar significant clashes of the Gal residue with the protein as observed with the A antigen (Fig. 4f).

To further support these findings at the molecular level, we first performed 0.5 µs molecular dynamics (MD) simulations of the FucOB in complex with H, A, and B type antigens obtained by in silico molecular docking calculations (see Fig. 4d–f, respectively). The relative movement between the ligands and the enzyme was analyzed by monitoring the distance between the center of the aromatic ring of W378 and the methyl group of the fucose residue (Supplementary Fig. 8). The MD simulations clearly show that the type H antigen is stable and maintains this interaction throughout the trajectory. Several hydrogen bonds between the antigen and FucOB are also formed. In contrast, the type A and B antigens explore other areas of FucOB or almost detach from the enzyme (Supplementary Fig. 8).

**Fig. 5:**
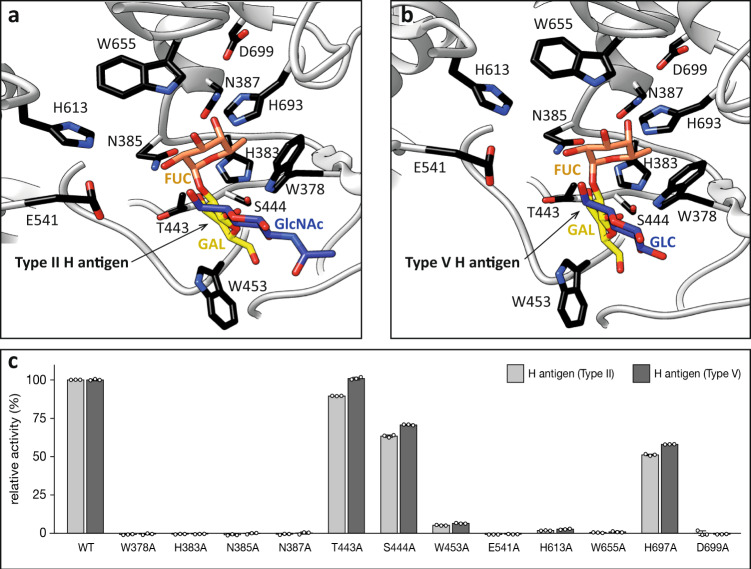
Alanine scanning mutagenesis of the FucOB-H antigen interface. **a**, **b** Structure of the FucOB (gray) complex with Type II H-antigen (Fuc in orange; Gal in yellow; GlcNAc in blue; panel **a**) and Type V H-antigen (Fuc in orange; Gal in yellow; Glc in blue; panel **b**) in which the amino acids mutated by alanine are in black. **c** Amount of fucose released by FucOB and FucOB mutants, from Type II H-antigen and Type V H-antigens. All values are shown relative to the wild-type enzyme. All enzymatic activity measurements were determined in triplicates (shown as three empty dots). Bars and error bars represent the mean and standard deviation of triplicate measurements. Source data are provided as a Source Data file.

We then performed single-point alanine mutations of residues in the loops that decorate the β-barrel core of the enzyme and contact the H antigen substrate (Fig. 5). We studied the ability of 11 single-point mutants to process the fucose residue on two substrates, Type II and Type V H antigens. Specifically, we individually mutated key residues in loop 25 (W378A, H383A, N385A), loop 28 (T443A, S444A, W453A), loop 35 (H613A); loop 37 (W655A), loop 39 (H693A and D699A) and α-helix 8 (N387A), in addition to the catalytically inactive E541A (Fig. 5a, b; Supplementary Fig. 9). Collectively, the mutational analysis of the FucOB loops that contact the fucose residue (loops 25, 35, 37, and 39) and N387A located in α-helix 8, indicated they were critical for Type II and Type V H antigens recognition (Fig. 5c). Interestingly, the mutations T443A and S444A were nearly dispensable for the enzymatic activity, while W453A resulted in a very significant reduction of the FucOB activity (Fig. 5c). It is worth mentioning that residues T443, S444, and W453 of loop 28 contacts the galactose ring. The side chains of T443 and S444 make hydrogen bonds with O3 and O4 atoms of galactose, while W453 stabilizes the sugar ring by a stacking interaction which, in light of the experimental data, seems to play an important role in FucOB substrate binding (Fig. 5a–c).

A detailed comparison of FucOB structures with that of the only four GH95 members for which experimental structural information is currently known provides additional deep insights into the molecular mechanism of H-type blood group epitope recognition and specificity. The three-domain architecture is essentially preserved between the five GH95 members, with FucOB displaying the largest substrate binding groove (Supplementary Figs. 5 and 10). However, the structural variability of the loops that decorate the active site might account for the differences in enzymatic activity and substrate specificity. In that sense, the available structural information of enzyme-ligands complexes is also limited to just four: α-L-galactosidase BACOVA_03438 in the presence of β-L-Gal (PDB code 4UFC), and α-1,2-fucosidase *Bb*AfcA in the presence of the substrate 2’FL (PDB code 2EAD), the products α-L-Fuc and Galβ1–4Glc (PDB code 2EAE) and the deoxyfuconojirimycin inhibitor (DFJ; PDB code 2EAC). The active site along the GH95 family shows two different conformations of the loop where the catalytic E541 is located. In the unliganded structures of FucOB and *Bb*AfcA (PDB code 2EAB), this loop moves away from the binding site creating an open groove. However, this loop adopts a different conformation in GH95 family crystal complexes of *Bb*AfcA (PDB code 2EAC and 2EAE) and BACOVA_03438 (PDB code 4UFC) with glycan substrates, moving around 2.8 Å closer to the substrate and closing the active site.

Structural comparison of the FucOB complex with the H-type antigen obtained by in silico molecular docking calculations with that of *Bb*AfcA in complex with 2’FL reveals that both ligands superimpose well (Supplementary Fig. 11). Single-point mutational analyses in the FucOB and *Bb*AfcA enzymes support that residues N385, N387, E541, and D699 (N421, N423, E566, and D766 in *Bb*AfcA, respectively) play critical roles in the catalytic mechanism of GH95 members. FucOB residues W378, H383, H613, W655, and H693, which also comprise the fucose binding pocket, were found essential for the enzymatic activity and are largely conserved not only in *Bb*AfcA (W414, H419, H678, W722, and H760, respectively) but also other α-1,2-fucosidase members of the GH95 family (Supplementary Fig. 5). Our mutagenesis data identified W453 as an important residue for the interaction with the galactose residue at the +1 subsite. Interestingly, the structure-based alignment shows variability in the region comprising loop 28. However, the enzymes preserve an aromatic residue at this position (W500 in *Bb*AfcA, F435 in BACOVA_03438; Supplementary Figs. 5 and 11). Altogether our structural and enzymatic data strongly support common substrate binding and catalytic mechanisms for the α-1,2-fucosidase members of the GH95 family.

Finally, a structural comparison between BACOVA_03438 (α-L-galactosidase) and *Bb*AfcA (α-1,2-L-fucosidase) suggested that a single residue located at the −1 subsite could determine the substrate specificity for L-galactose or L-fucose. Specifically, T370 in BACOVA_03438 interacts with the l-galactose O6 atom by hydrogen bonds, whereas H419 in *Bb*AfcA interacts with the L-fucose C6 methyl group by aliphatic interactions^35^. Nevertheless, very limited structural data are available for this family to conclude that this polymorphic position at the −1 subsite is the sole structural feature that determines the substrate specificity of this family. In that sense, subsequent biochemical characterization and the structural determination of other GH95 family members demonstrated that this polymorphic position was not essential for substrate recognition in this family of enzymes. This is the case of *Xac*Afc95 from *Xanthomonas axonopodis pv. citri*^34,37^, and Blon_2355 from *Bifidobacterium longum* subsp. *Infantis*^38^, both of which contain a threonine residue at −1 subsite and showed α-1,2-L-fucosidase activity (Supplementary Fig. 11). In addition, the mutation T395H in *Xac*Afc95 maintains the substrate specificity for L-fucose^34^. Interestingly, the catalytic domain of *Bb*AfcA did not liberate fucose from any of the artificial substrates examined, *p*-nitrophenyl (pNP)-α-L-fucoside, pNP-β-l-fucoside, and 4-methylumbelliferyl-α-L-fucoside^30^. Similarly, BACOVA_03438 lacks enzymatic activity against 4-nitrophenyl-α-L-galactopyranoside^35^. Therefore, it was suggested that the subsite +1 could also play a role in defining the enzymatic activity and specificity of GH95 enzymes^35^. However, according to our activity assays, FucOB and *Xac*Afc95^34^, hydrolyze fucose from pNP-α-L-fucoside artificial substrate. These findings support a more elaborate mechanism of substrate selectivity in the GH95 family members that is not restricted to direct interactions with the residues comprising the −1 subsite.

### FucOB converts universal O to rare Bombay-type blood group

**Fig. 6:**
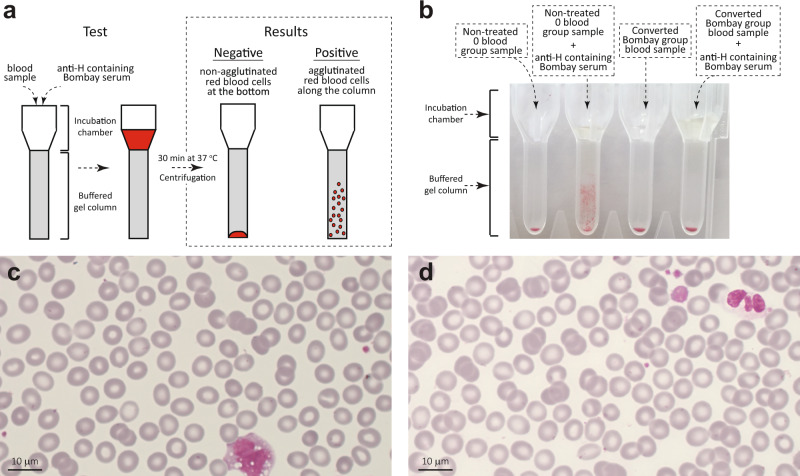
Turning universal O into rare Bombay-type blood. **a** Schematic representation of agglutination assay with DG Gel cards from Grifols (Diagnostic Grifols, S.A.) and positive and negative results representation. **b** Agglutination experiment results picture; negative/non-agglutination in non-treated O blood group sample in the first column and positive/agglutination in the second column in non-treated O blood group sample in contact with H antibodies naturally containing Bombay serum as a negative and positive control, respectively; negative/non-agglutination in the third column in converted Bombay blood group sample; negative/non-agglutinated result in converted Bombay blood group sample in contact with H antibodies naturally containing Bombay serum. **c** Non-treated O blood group smear picture. RCBs are shown with normal biconcave morphology. This experiment was performed on two different samples. **d** Converted O blood group to Bombay sample’s smear picture. RCBs are shown to maintain normal biconcave morphology. This experiment was performed on two different samples.

Our enzymatic activity assays clearly showed that FucOB can hydrolyze the α-1,2 linked fucose residue in Type I, Type II, Type III, and Type V H antigens, generating the afucosylated Bombay phenotype. In contrast, FucOB cannot cleave the α-1,2 linked fucose residue from Type V A antigen or the Type V B antigen. To determine whether FucOB can convert universal O into a rare Bombay type blood group, type O RBCs were incubated with 200, 50, 5, 0.5, 0.05, and 0.005 µg mL^−1^ of either FucOB or the catalytically inactive version of the enzyme FucOB_E541A_, and analyzed by agglutination assays against naturally containing anti-H Bombay serum (Fig. 6a, b; Supplementary Fig. 12; see the “Methods” section). Strikingly, as depicted in Fig. 6b, RBCs pre-incubated with FucOB at 200, 50, and 5 µg mL^−1^ showed no agglutination in the presence of Bombay serum that contains anti-H antibodies. This result clearly indicates the cleavage of the l-fucose present in the H antigen and therefore, the conversion of universal O into a rare Bombay-type blood group. It is worth noting that all O RBCs previously incubated (i) without enzyme or (ii) with the inactive FucOB_E541A_, agglutinated due to the presence of anti-H antibodies in the Bombay serum, showing the classic hemolytic reaction described once Bombay blood is mixed with any kind of ABO blood group sample^17^. To check whether this phenomenon is reproducible and representative in a larger number of samples, we repeated the experiment with RBCs from 20 donors, 10 O Rh negative and 10 O Rh positive. All universal O RBCs were converted to rare Bombay type blood group (Supplementary Fig. 13). To further support and validate our findings, we performed a secondary agglutination test using an anti-H lectin, a blood grouping reagent prepared from an extract of *Ulex europaeus* seeds. As depicted in Supplementary Fig. 14, RBCs from O donors pre-incubated with FucOB 5 µg mL^−1^ showed no agglutination in the presence of the anti-H lectin. In contrast, the catalytically inactive FucOB_E541A_ showed a clear agglutination reaction after treatment with the anti-H lectin. These results also support the cleavage of L-fucose present in the H antigen by FucOB and the conversion of universal O into a rare Bombay-type blood group.

The viability and integrity of converted Bombay RBCs by the action of FucOB were essentially preserved (Fig. 6; Supplementary Fig. 15). Supporting this notion, RBCs belonging to O-negative and O-positive blood groups were subjected to different tests that are carried out routinely in the clinic. On the one hand, RBCs from non-treated blood, as well as RBCs incubated with and without 50 µg mL^−1^ of FucOB at 37 °C, were visualized in blood smears to check whereas the morphology of the erythrocytes is affected by (i) the incubation temperature or (ii) the action of FucOB on the H surface antigen of the erythrocytes. As shown in Fig. 6c,d, all RBCs display a normal biconcave morphology^39^. On the other hand, we performed the glucose-6-phosphate dehydrogenase (G6PD) assay in the same RBCs. G6PD is a ubiquitous enzyme present in the membrane of erythrocytes that plays a critical role in the redox metabolism of all aerobic cells. The enzyme catalyzes the first and rate-limiting step of the pentose phosphate pathway, generating NADPH and ribose-5-phosphate, which is essential for the production of nucleotide coenzymes and nucleic acids and therefore, cell division^40^. The positive results in all of our samples indicate that the viability and integrity of the cell membrane are preserved (Supplementary Fig. 15).

**Fig. 7:**
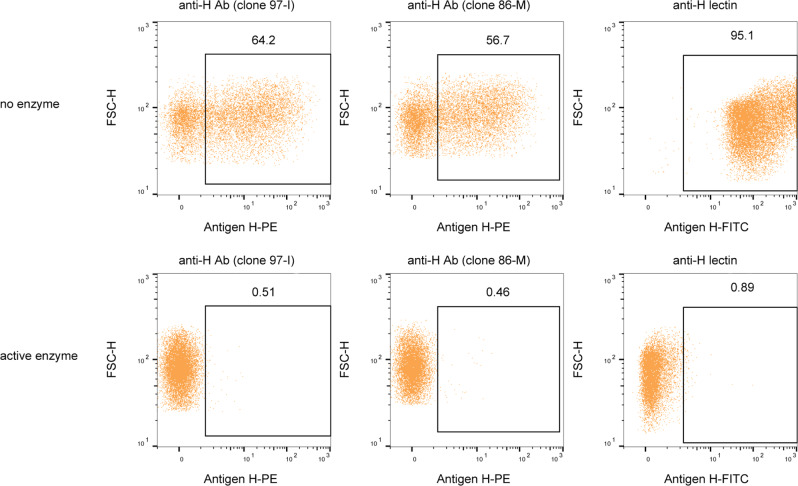
Enzymatic blood type conversion as determined by flow cytometry. Different labeling strategies for the analysis of antigen H expression in RBCs. Dot plot graphs showing the frequency of antigen H+ RBCs from an O blood group sample donor non-incubated and incubated with the active FucOB (5 µg mL^−1^ final concentration). Two different antibodies (clone 97-I left column panels and clone 86-M middle column panels) and an antigen H-specific lectin (right column panels) were used to detect antigen H+ RBCs.

To evaluate whether the conversion of O-type RBCs by FucOB was complete, we studied by fluorescence-activated cell sorting (FACS) analysis after treatment of O-type RBCs with the active enzyme (Fig. 7). For that, we first identified RBCs based on the expression of CD235a (glycophorin A), a transmembrane glycoprotein expressed by erythrocytes, and then determine the frequency of antigen H+ cells (see the gating strategy in Supplementary Fig. 16) with 2 different anti-H blood group monoclonal antibodies (clone 97-I and clone 86-M anti-H monoclonal antibodies) and an H antigen recognizing conjugated lectin (see the “Methods” section). The percentages of positive cells resulted in (i) 64.2% when we used clone 97-I, and (ii) 56.7%, when we used clone 86-M. Importantly, the experiments performed with the anti-H lectin showed that practically all group O red cells, 95.1%, were positive. It is worth noting that the inability of anti-H antibodies to label all O-type RBCs may be related to the fact that the expression of blood antigens is variable depending on the donors^41,42^ and/or the quality of the antibodies. Interestingly, in the literature, the expression of the H antigen by flow cytometry on O-type RBCs is generally performed by staining with anti-H lectin^43^. Importantly, the binding of the anti-H monoclonal antibodies and the anti-H lectin completely disappeared when O-type RBCs were pre-treated with FucOB (Fig. 7).

## Discussion

The only structural difference between the ABO blood group antigens is the presence or absence of an external sugar residue linked to the common chain precursor in A, B, and H antigens, respectively (Fig. 1)^15^. Therefore, the enzymatic modification of one sugar in the oligosaccharide could change the blood group of RBCs into another one, being the conversion of A, B, and AB blood groups into O, the called universal blood, highly important for the universal blood supply of blood banks in emergency situations^44^. This concept was first proposed/demonstrated using an α-galactosidase from green coffee beans, to convert B-type RBCs into universal O, following a subsequent successful transfusion^45,46^. Two families of α-*N*-acetylgalactosaminidases (GH109) and α-galactosidases (GH110) successfully converted their corresponding B and A RBCs^47^. However, large quantities of enzymes were needed, rendering the approaches impracticable. The complete removal of both the A and B antigens was achieved with the endo-galactosidase E-ABase from *Clostridium perfringens*, which cleaves the terminal trisaccharides^48^. More recently, a significant advance was made by functional metagenomic screening of the human gut microbiome for enzymes that can convert the A or B-type antigens into universal O antigens. An enzymatic pathway from the obligate anaerobe *Flavonifractor plautii* comprising (i) an *N*-acetylgalactosamine deacetylase and (ii) a galactosaminidase (GH36), converted A^+^ RBCs to O-type universal donor RBCs via a unique mechanism^49^. Their ability to complete the conversion at very low enzyme concentrations in whole blood will simplify their incorporation into blood transfusion practice, broadening blood supply^49^. In addition, both enzymes were also used to convert blood group A lungs to blood group O lungs using ex vivo lung perfusion^50^. The authors showed minimized antibody binding, complement deposition, and antibody-mediated injury after enzymatic treatment suggesting that this strategy has the potential to improve equity in the allocation of organs for transplantation^50^.

A rare blood donor phenotype occurs at least 1/1000 and includes high-frequency-antigen-negative and multiple-common-antigen-negative blood groups, including the Bombay phenotype. Due to the low frequency of each rare blood group, it is crucial to have an accurate international database of rare blood donors to ensure that patients who require lifesaving rare blood units can receive them. There is also a rare inherited primary immunodeficiency disorder called leukocyte adhesion deficiency (LAD) that causes the Bombay phenotype. Type II (LAD II) disorder results from a defect in fucose metabolism which enables the correct fucosylation of glycoproteins, including H antigen biosynthesis^51,52^. Due to the low prevalence of each rare disease, medical expertise is rare, knowledge is scarce, care offerings inadequate, and research is limited. Thus, despite their large overall number, rare disease patients are the orphans of health systems^53,54^.

Efforts were concentrated on the identification of an enzyme capable of performing the conversion of universal type H into rare Bombay-type blood. Up to date, only 14 GH95 family members were reported to have α-1,2-L-fucosidase activity (EC 3.2.1.63; Fucα1-2Galβ as substrate; 11 from bacterial species and three eukaryotic enzymes; Supplementary Table 1). The removal of the fucose residue from the H-antigen oligosaccharide in RBCs was initially explored using an α-1,2-fucosidase from *Aspergillus niger*^55^. However, the enzyme displayed an optimum pH of 4.5, limiting its practical application^56^. An α-1,2-fucosidase successfully modified the Type II chain H antigen on RBCs. However, the Type III chain H antigen was unaffected. In addition, the enzyme displayed maximum activity at acidic pH values, reducing its application^57^. Interestingly, synthetic metallopeptides of 16 to 20 amino acids were designed as artificial fucosidases to remove fucose from Type II H antigen on RBCs^58^. More recently, a membrane α-1,2-fucosidase from *Elizabethkingia meningoseptica* showed activity on Type I, Type II, and Type IV H antigens, producing H-deficient RBCs^59^. Its amino acid sequence displayed ca. 23.9% identity with the soluble FucOB from *A. muciniphila*^60^. FucOB shows encouraging characteristics as an α-1,2-fucosidase. First, FucOB is easily produced in high yields and purity, 5.0 mg L^−1^. Second, according to our Bombay conversion enzymatic assays, the amount of active enzyme able to hydrolyze the fucose from H antigen in O blood samples is very low, 5 µg mL^−1^. Finally, FucOB displayed the ability to convert O group into Bombay in complete blood and non-washed RBC samples. We directly incubated the enzyme at 37 °C with blood samples collected from the Blood Bank to perform G6PD activity assay and blood smears and we measured that the enzyme can hydrolyze the fucose from H antigens in that condition. Altogether, we propose FucOB as a promising biotechnological and therapeutic tool to cleave the fucose present in O-type blood RBCs and therefore, convert universal O-type blood into rare Bombay-type blood facilitating the transfusion to Bombay phenotype individuals. Taking into account our extensive/thorough structural, biochemical, and substrate specificity analysis of FucOB, it is tempting to speculate that other members of the GH95 family (e.g. *Bb*AfcA) will likely serve the same purpose as FucOB regarding the conversion of O blood group to the Bombay blood group.

Given the complexity and diversity of intestinal mucin glycan structures, the deconstruction of these molecules involves the concerted action of GHs encoded by the genome of mucin-degrading bacteria. In mucins, the core structures are elongated and frequently modified by fucose residues through α-1,2, α-1,3, and α-1,4 linkages^9,61^. Genes encoding for fucosidases are widely distributed in the genome of gut bacteria. They mostly belong to the GH29 and GH95 families, which follow retaining and inverting catalytic mechanisms^33,62^, respectively. Transcriptional data support the notion that GH29 and GH95 fucosidases play an important role in the ability of *Bacteroides thetaiotaomicron* VPI-5482^63^, *Bifidobacterium longum* subsp. *infantis* ATCC 15697^38^, *Bifidobacterium bifidum* JCM 1254^64^, and *Ruminococcus gnavus* ATCC 29149^65^ to utilize mucins as a source of carbon. To date, only four GH95 fucosidases from human gut commensal strains have been biochemically characterized: (i) α-1,2-L-fucosidase AfcA from *B. bifidum* JCM 1254^30^, (ii) α-1,2-L-fucosidase Blon_2335 from *B. longum* subsp. *infantis* ATCC 697^38^, (iii) α-1,2-fucosidase *Ri*Fuc95 from *Roseburia inulinivorans* DSM 16841^66^, and (iv) α-L-fucosidase RUMGNA_00842 from *R. gnavus* ATCC 29149^67^. The genome of *A. muciniphila* strain ATCC BAA-835 encodes four GH29 and two GH95 genes. Both GH95 fucosidases Amuc_0186 and FucOB (Amuc_1120) were significantly upregulated when *A. muciniphila* was grown in mucin^32^. Although there is no experimental evidence of Sus-like systems in Verricomicrobium phylum^26^, careful inspection of the protein-encoding genes in the genome of *A. muciniphila* ATCC BAA-835 strain showed that the *fucOB* gene is close to (i) OgpA, a paradigmatic *O*-glycopeptidase that exclusively hydrolyzes the peptide bond N-terminal to serine or threonine residues substituted with an *O*-glycan, and (ii) a predicted sulfatase (Amuc_1118) (Supplementary Fig. 1). Altogether, our experimental data support that the two enzymes seem to be part of a conserved cluster dedicated to the mucin degradation system.

The composition and physiology of the gut microbiota play a major role in human health and disease. Alterations of such equilibrium have been implicated in several pathologies, including metabolic disease, cardiovascular disease, type-2 diabetes, and cancer^23,24,68^. In the colon, the mucus lining the epithelium is critical for maintaining a homeostatic relationship with the gut microbiota by harboring a microbial community at a safe distance from the epithelial surface^69^. The mucin glycans that make up the mucus layer provide binding sites and a sustainable source of nutrients for bacteria that inhabit the mucus niche^70^. The peripheral terminal epitopes show considerable variation with a decreasing gradient of fucose and ABH blood group antigens expression from the ileum to the colon^71^. For example, H and A blood group antigens were shown to be present exclusively in the ileum and cecum^72^. It is worth noting that ABH blood group antigens can play a direct role in infection by serving as receptors and/or co-receptors for bacteria, parasites, and viruses^73^. Interestingly, the human gut symbiont *R. gnavus* showed specificity to blood group A antigen during mucin glycan foraging. This capacity was conferred by a gene encoding for a predicted GH98 blood-group endo-β-1,4-galactosidase^67^. The experimental data point support that the GH repertoire of *R. gnavus* strains enables them to colonize different nutritional niches in the human gut, a model that could be operational in other members of the gut microbiota.

Carbohydrate-specific enzymes are well-established tools and are often used to advance the analytical methods within glycobiology. The use of high-resolution mass spectrometry in glycobiology research has increased both the sensitivity and the level of detail that can be studied. Many of the enzymatic tools available have remained the same since their development, often in the early days of glycobiology. Lately, several new and improved exoglycosidases have been brought to the market and found applications within basic glycobiology research and as tools for the biopharmaceutical industry developing novel glycoprotein therapeutics^29,74–76^. FucOB is commercialized in the context of an α-fucosidase mix, FucosEXO™, for efficient removal of α1-2, α1-3, and α1-4 linked fucose from *N*- and *O*-glycoproteins or free oligosaccharides and applications in fundamental glycomics and research^77^. This illustrates how novel enzymes such as FucOB can be used as tools to address scientific challenges and push the boundaries of science.

## Methods

### Materials

Blood group H antigen triaose type I, blood group H antigen triaose type V (2’-fucosyllactose), blood group H antigen triaose type II, blood group A antigen triaose type V, blood group B antigen triaose type V were purchased from ELICITYL. 3-fucosyllactose was purchased from Carbosynth, GDP-Fucose, and Lewis a trisaccharide from Sigma Aldrich, and 6-fucosylchitobiose (GlcNAcβ(1-4)[Fucα(1-6)]GlcNAc) from TCI. Anti-blood group H ab antigen antibody [97-I] (ab24213; the epitope is a carbohydrate moiety) and mouse IgM [B11/7]-Isotype control (ab91545) were purchased from Abcam, anti-blood Group H n/ab antigen antibody [86-M] (AGM-022YJ) was purchased from Creative Biolabs, goat anti-mouse IgM (Heavy chain) cross-adsorbed secondary antibody PE (M31504) and CaptureSelect IgG-Fc were purchased from Thermo Fisher Scientific, and BV421 Mouse Anti-Human CD235a were purchased from BD Biosciences (cat. number 562938) Anti-H lectin reagent (extract of *Ulex europaeus* seeds) was purchased from BioRad and FITC-conjugated anti-H lectin (also an extract of *Ulex Europeaus*) (L32476) was purchase from Thermo Fisher Scientific. SialEXO, PNGase F, and FabRICTOR were from Genovis AB and Recombinant Fut2 was purchased from R&D systems. Fucosidase 95A from *Bifidobacterium longum* (CZ0511) was purchased from NZYTech.

### Human samples and ethics statement

Tubular segments attached to RBCs concentrate units from anonymous healthy donors were obtained from the Blood Bank of Cruces University Hospital. The study was approved by the Ethics Committee for Clinical Research of Cruces University Hospital (CEI E21/65) in accordance with Spanish Law and the Declaration of Helsinki.

### Cloning of wild-type and single-point mutants of FucOB from *A. muciniphila strain* ATCC BAA-835 and *Bb*AfcA

The pET29a-*Amuc_1120*, pET29a-*Amuc_*1120^E541A^ (pET29a-*fucOB* and pET29a-*fucOB*^E541A^ genes, hereafter), and the catalytic domain of pET29a-*BbAfcA*^33^ were synthesized/sequenced by ATG: biosynthetic. The *fucOB*, *fucOB*^E541A^, and *BbAfcA* were introduced into the pET29a plasmid using the NdeI and HindIII sites. The recombinant FucOB and FucOB_E541A_ (796 residues) have a deletion of the first 23 residues that were predicted as a signal peptide and an additional peptide of 17 amino acids at the N-terminus that includes a histidine tag (Supplementary Fig. 2). pET29a-*fucOB*^W378A^, pET29a-*fucOB*^H383A^, pET29a-*fucOB*^N385A^, pET29a-*fucOB*^N387A^, pET29a-*fucOB*^T443A^, pET29a-*fucOB*^S444A^, pET29a-*fucOB*^W453A^, pET29a-*fucOB*^H613A^, pET29a-*fucOB*^W655A^, pET29a-*fucOB*^H693A^, pET29a-*fucOB*^D699A^ were generated by QuikChange site-directed mutagenesis (Fig. 5; Supplementary Figs. 9 and 11; Supplementary Table 3)^78^. *BbAfcA* also includes a histidine tag at N-terminus.

### Expression and purification of wild-type and single-point mutants of FucOB from *Akkermansia muciniphila strain* ATCC BAA-835 and *Bb*AfcA

*Escherichia coli* BL21 (DE3) cells transformed with pET29a-*fucOB*, pET29a-*fucOB*^E541A^, pET29a-*fucOB*^W378A^, pET29a-*fucOB*^H383A^, pET29a-*fucOB*^N385A^, pET29a-*fucOB*^N387A^, pET29a-*fucOB*^T443A^, pET29a-*fucOB*^S444A^, pET29a-*fucOB*^W453A^, pET29a-*fucOB*^H613A^, pET29a-*fucOB*^W655A^, pET29a-*fucOB*^H693A^, pET29a-*fucOB*^D699A^ or pET29a-*BbAfcA* were grown in Luria Broth (LB) medium supplemented with 50 μg mL^−1^ of kanamycin at 37 °C. When the culture reached OD_600_ = 0.6, Protein expression was induced by adding 1.0 mM isopropyl β-thiogalactopyranoside (IPTG). After 20 h at 18 °C, cells were harvested at 5000 × *g* for 20 min at 4 °C and resuspended in 50 mL of 50 mM Tris-HCl pH 7.5, 500 mM NaCl, containing protease inhibitors (Complete EDTA-free, Roche) and 0.5 µL L^−1^ of the culture of benzonase (Sigma Aldrich). Cells were then disrupted by sonication in 12 cycles of 10 s pulses, with 60 s cooling intervals between the pulses, and 60% of amplitude and the suspension was centrifuged for 30 min at 59,000 × *g* at 18 °C. The supernatant was filtered by 0.22 μm pore size Merck Millipore Durapore™ PVDF Membrane Filters and subjected to Ni^2+^-affinity chromatography using a HisTrap Chelating column (5 mL, GE HealthCare) equilibrated in 50 mM Tris-HCl pH 7.5, 500 mM NaCl. Elution was performed with a linear gradient of 0–500 mM imidazole in 300 mL of 50 mM Tris-HCl pH 7.5, 500 mM NaCl at 4 mL min^−1^. Fractions of interest were pooled, buffer exchanged to 50 mM HEPES pH 7.0 in a 30 kDa cut-off centrifugal filter, and loaded into a HiTrap SP HP column (1 mL; GE HealthCare), equilibrated in buffer 50 mM HEPES pH 7.0. Elution was performed with a linear gradient of 0–1000 mM NaCl in 30 mL of 50 mM HEPES pH 7.0, at 1 mL min^−1^. Fractions of interest were pooled and loaded onto a Superdex 75 16/600GL (GE Healthcare) equilibrated in the corresponding buffer according to the experiment to be performed (20 mM Tris-HCl pH 7.5. for crystallization assays and 50 mM Tris-HCl pH 7.5 and 150 mM NaCl for blood group conversion assays). Fractions of interest were pooled and concentrated to 14 mg mL^−1^ and 15 mg mL^−1^, respectively, in 20 mM Tris-HCl pH 7.5, using a 30 kDa cut-off centrifugal filter (Millipore) for crystallization purposes. The resulting preparations displayed a single protein band by SDS-PAGE (Supplementary Figs. 2 and  9). Purified FucOB and FucOB_E541A_ were produced at 5.0 and 6.8 mg L^−1^ of growth culture. Purified proteins were stored at −80 °C.

### FucOB, *Bb*AfcA and *Bl*Fuc95A substrate specificity assays

120 nmol each of 3-fucosyllactose, Lewis-A trisaccharide, 6-fucosylchitobiose, blood group H antigen triaose Type I, Type II, and Type V (2’-fucosyllactose), blood group A antigen tetraose Type V or blood group B antigen tetraose Type V were incubated with FucOB, *Bb*AfcA, and *Bl*Fuc95A, at a molar enzyme to substrate ratio of 1:100,000 in 20 mM Tris-HCl pH 6.8. After 30 min incubation at 37 °C, the reactions were stopped by heating to 90 °C for 10 min and the amount of released fucose in each reaction was determined using the L-fucose assay kit (Megazyme) according to the manufacturer’s instructions. In short, L-fucose dehydrogenase and NADP^+^ were added to the FucOB, *Bb*AfcA, and *Bi*Fuc95A digested samples, and the formation of NADPH during oxidation of l-fucose, stoichiometric with the amount of free l-fucose in the sample, was monitored spectrophotometrically.

### FucOB activity assay using *p*-nitrophenyl-α-l-fucose (pNP-Fuc)

500 nmol pNP-Fuc was incubated with 40 µg FucOB in 50 µL 20 mM Tris-HCl pH 6.8 at 37 °C for 4 h. The reaction was stopped by the addition of 0.1% formic acid and the product was separated from the educt by reverse-phase HPLC and quantified using UV detection at 300 nm.

### Glycoengineering of TNFR

1 mg etanercept (TNFR/IgG1 Fc fusion protein) was incubated with 1000 u SialEXO and 1000 u PNGase F for 4 h at 37 °C to desialylate the *O*-glycans and remove the *N*-glycans (to simplify the analysis). 8 mM GDP-fucose, 10 mM MnCl_2_, 10 mM CaCl_2_, and 3 μg recombinant human Fut2 fucosyltransferase were added and the resulting mixture was incubated at 37 °C overnight. The resulting fucosylated glycoprotein was purified using CaptureSelect IgG-Fc (multispecies) according to the manufacturer’s recommendations.

### FucOB activity assays by LC-MS

20 μg of fucosylated etanercept was incubated with 1 μg FucOB for 1 h at 37 °C in a total volume of 40 μL 20 mM Tris pH 6.8. To simplify the analysis, the TNFR domain carrying the fucosylated *O*-glycans was separated from the Fc region by digestion with FabRICATOR (IdeS) protease that cleaves the IgG Fc fusion protein at one specific site below the hinge. The resulting protein subunits were reduced and denatured by incubation for 1 h at 37 °C in 4 M guanidine-HCl, 100 mM DTT and analyzed by reverse phase LC-MS using a Bruker Impact II ESI-QTOF mass spectrometer.

### Activity assay of FucOB mutants

120 nmol each of blood group H antigen triaose type II and V were incubated with FucOB at a molar enzyme to substrate ratio of 1:50 000 in 20 mM Tris-HCl pH 6.8. After 30 min incubation at 37 °C, the reactions were stopped by addition of 1:6 (v:v) 1 M Tris-HCl pH 10 and the amount of released fucose in each reaction was determined using the L-fucose assay kit (Megazyme) according to the manufacturer’s instructions.

### FucOB and FucOB_E541A_ crystallization and data collection

FucOB was crystallized by mixing 0.25 µL of a protein solution at 14 mg mL^−1^ in 20 mM Tris-HCl pH 7.5 with 0.05 µL of seed stock solution and 0.2 µL of 200 mM potassium fluoride, 20% (w/v) PEG 3500 (PEG ION HR2-126 protein crystallization screen, Hampton Research). Seeds stock solution was prepared following Hampton Research protocols and vortexed bead seed stock technique procedures^79^. Crystals grew in 120 days and were cryo-cooled in liquid nitrogen using 200 mM potassium fluoride, 20% (w/v) PEG 3500, and 20% glycerol, as cryo-protectant solution. The FucOB_E541A_ was crystallized by mixing 0.25 µL of a protein solution at 15 mg mL^−1^ and 2.5 mM of A type V blood antigen in 20 mM Tris-HCl pH 7.5 with 0.05 µL of seed stock solution and 0.2 µL of 200 mM sodium chloride, 20% (w/v) PEG 3350 (PEG ION suite protein crystallization screening, Hampton Research). Crystals grew in 15 days and were cryo-cooled in liquid nitrogen using 200 mM sodium chloride 20% (w/v) PEG 3350 and 20% glycerol, as cryo-protectant solution. Complete X-ray diffraction datasets for FucOB and FucOB_E541A_ were collected at X06DA-PXIII beamline, at the Swiss Light Source, the Paul Scherrer Institute, Switzerland, and BL13-XALOC beamline at ALBA, Cerdanyola del Valles, Spain, respectively. FucOB crystallized in the orthorhombic space group *P* 2_1_ 2_1_ 2_1_ with one molecule in the asymmetric unit and diffracted to a maximum resolution of 1.8 Å (Supplementary Table 1). FucOB_E541A_ crystallized in the monoclinic space group *P* 2_1_ with one molecule in the asymmetric unit and diffracted to a maximum resolution of 1.95 Å (Supplementary Table 1)^80^. All datasets were integrated and scaled with XDS following standard procedures^81^.

### FucOB and FucOB_E541A_ structure determination and refinement

The structure determination of FucOB and FucOB_E541A_ was carried out by molecular replacement methods implemented in Phaser^82^ and the PHENIX suite^83^ using the PDB code 2EAB as a search template. Initial cycles of model building, density modifications, and refinement by Buccaneer^84^ and the CCP4 suite^82^. The final manual building was performed with Coot^84^ and refinement with phenix refine^85^. The structures were validated by MolProbity^86^. Data collection and refinement statistics are presented in Supplementary Table 1. The atomic coordinates and structure factors were deposited with the Protein Data Bank, accession codes are 7ZNZ and 7ZO0. Molecular graphics and structural analyses were performed with the UCSF Chimera package^87^.

### Structural analysis and sequence alignment

Structure-based sequence alignment analysis was performed using Chimera^87^. Protein pocket volume was calculated using HOLLOW^88^. *Z*-score values were produced by using DALI^89^. Domain interface analysis was performed using PISA^90^. Conserved and similar residues were labeled using the Multiple Align Show server (https://www.bioinformatics.org/SMS/multi_align.html).

### Molecular docking calculations

H, A, and B antigens were modeled using GLYCAM-Web website (Complex Carbohydrate Research Center, University of Georgia, Athens, GA; http://www.glycam.com). Ligand docking was performed using AutoDock Vina employing standard parameters^91^ and visualized using USCF Chimera^87^. The active site of FucOB was defined taking into account the crystal structure of the homologous α-1,2-fucosidase *Bb*AfcA in complex with the substrate 2’-fucosyllactose (2’FL; Fucα1-2Galβ1-4Glc; PDB code 2EAD).

### Molecular dynamics (MD) simulations

We used the in silico molecular docking structures shown in Fig. 4 as the initial structures for the three complexed studied by MD simulations. The simulations were carried out with AMBER 20 package^92^ implemented with ff14SB^93^ and GLYCAM06j^94^ force fields. The system was neutralized by adding explicit counter ions. Each complex was immersed in a water box with a 10 Å buffer of TIP3P water molecules^95^. A two-stage geometry optimization approach was performed. The first stage minimizes only the positions of solvent molecules, and the second stage is an unrestrained minimization of all the atoms in the simulation cell. The systems were then gently heated by incrementing the temperature from 0 to 300 K under the constant pressure of 1 atm and periodic boundary conditions. Harmonic restraints of 30 kcal mol^−1^ were applied to the solute, and the Andersen temperature coupling scheme was used to control and equalize the temperature. The time step was kept at 1 fs during the heating stages, allowing potential inhomogeneities to self-adjust. Long-range electrostatic effects were modeled using the particle-mesh-Ewald method^96^. An 8 Å cut-off was applied to Lennard-Jones interactions. Each system was equilibrated for 2 ns with a 2-fs time step at a constant volume and temperature of 300 K. Production trajectories was then run for additional 0.5 µs under the same simulation conditions.

### Blood samples extraction, collection, and storage

Samples of RBC concentrates from anonymous healthy donors were obtained from blood bag tubing segments at the Blood Bank of Cruces University Hospital. The samples containing standard preserving CPD-SAGM media^97^ were stored at 4 °C until used for up to 35 days. RBCs from non-treated blood were collected the same day of the experiment from two healthy consenting donors into a citrate Vacutainer using a protocol approved by the Ethics Committee for Clinical Research of Cruces University Hospital (CEI E21/65) in accordance with the Spanish Law and the Declaration of Helsinki.

### Enzymatic conversion of universal O into rare Bombay-type blood group assay

To analyze the enzymatic conversion of universal O into rare Bombay type 2 mL of healthy donors’ blood samples were first diluted with 18 mL of PBS to reach a 4% red cells suspension and washed twice with PBS (centrifuged at 5000 × *g* for 5 min at 4 °C). After removing the supernatant, 18 mL of PBS was added and the suspension was separated into 18 aliquots of 1 mL final volume. The samples were centrifuged at 200 × *g* for 5 min at RT. After removing the supernatant, the needed amount of enzyme was added over 200 μL final sample volume, to reach a final concentration of 200, 50, 5.0, 0.5, 0.05, and 0.005 μL mL^−1^ of FucOB or FucOB_E541A_. The mixtures were maintained in an incubator OPAQ I10-E and the orbital MaXI shaker OL30-ME (OVAN) at a constant stirring of 110 rpm for 30 min at 37 °C. The cells were then washed twice with 1 mL of PBS. Finally, each sample was diluted in 200 μL of PBS per sample.

### DG Gel column agglutination assay

DG Gel Neutral and DG Gel Coombs cards from Grifols (Diagnostic Grifols, S.A.) were used for blood group typing and agglutination assays based on the gel technique described in 1985 by Ives Lapierre^98^. We followed the manufacturer’s recommendations. 10 μL of RBC sample was diluted in 1 mL of Grifols Diluent. 25 μL of Bombay serum was added to the selected DG Gel wells and 50 μL of previously diluted RBC sample was then added. The DG Gel Neutral cards were incubated for 15 min at RT, whereas DG Gel Coombs cards were incubated for 15 min at 37 °C, and later centrifuge in DG SPIN centrifuge for 9 min at RT. RBCs that underwent agglutination with anti-H antibody present in Bombay serum were evaluated following the manufacturer’s instructions. Briefly, the presence of an RBCs pellet in the bottom of the gel column indicates no agglutination (negative result) neither hemolysis in the sample. On the other hand, clumps of RBCs throughout the gel column indicate cells agglutinated in the sample (positive result). Each agglutination card has 8 buffered tubes to perform the experiments. Therefore, the results displayed in the corresponding Figures are shown in patches.

### Anti-H lectin agglutination assay

1 mL of two healthy O-negative blood group donor’s samples and 1 mL of one healthy B-positive blood group donor sample were first washed twice with 9 mL of PBS and centrifuged at 5000 × *g* for 5 min at 4 °C. The washed samples were diluted to 500 μL of 4% of red cells suspension. 5 μL of the needed amount of FucOB, FucOB_E541A_, or PBS (as a control) was added over the red cells suspension to reach a final concentration of 5.0 μg mL^−1^ of the enzyme. The mixtures were incubated for 10 min at 37 °C. 25 μL of each sample was added over 50 μL of anti-H lectin reagent or PBS (as a control) and results were read macroscopically. The presence of RBCs pellet in the bottom indicates no agglutination (negative result) or hemolysis in the sample. In contrast, a reddish solution of RBCs indicates cells agglutinated in the sample (positive result).

### Blood smears

To visualize the RBC physical preservation after conversion, treated and control samples were observed by May-Grünwald-Giemsa stained smears^99^. Specifically, 200 μL of O-negative blood samples were directly incubated with 50 μg mL^−1^ of active enzyme in an incubator OPAQ I10-E and the orbital MaXI shaker OL30-ME (OVAN) at the constant stirring of 110 rpm for 30 min at 37 °C. One drop of enzymatically converted blood sample was later spread in a glass slide and fixed by dipping it in absolute methanol for three minutes. Then, an equal volume of stain solution 1 (0.3 g May-Grünwald powder in 100 mL absolute methanol) was freshly mixed with a buffer solution at pH 6.8 of 6.63 g KH_2_PO_4_, 2.56 g Na_2_HPO_4_ 2H_2_O and distilled water up to 1000 mL. The mixture was applied over the fixed sample for 5 min horizontally positioned. A dilution of stain solution 2 (1 g Giemsa stain powder dissolved in 66 mL glycerol, and heated to 56 °C for 120 min to later add 66 mL of absolute methanol) in the same buffer solution (1:9; v/v) was added over the sample for 15 min. Finally, the stained sample was washed with water and visualized with an optical microscope.

### Glucose-6-phosphate dehydrogenase assay

To perform glucose-6-phosphate dehydrogenase activity assay 200 μL of O negative blood sample was directly incubated with 50 μg mL^−1^ of active enzyme in an incubator OPAQ I10-E and the orbital MaXI shaker OL30-ME (OVAN) at the constant stirring of 110 rpm incubator for 30 min at 37 °C. Trinity Biotech Glucose-6-Phosphate Dehydrogenase (G6PD) reagents were used to perform a G6PD deficiency assay in converted blood samples following the manufacturer’s instructions^40^. 0.2 ml of G6PD substrate solution (Glucose-6-Phosphate (4 μmol), NADP (1.6 μmol), Glutathione, oxidized (1.6 μmol), and lytic agent in 2 mL volume) were incubated at 37 °C with 0.01 mL blood sample.

### Flow cytometry studies

For FACS analysis, enzymatically treated O-type RBCs were diluted (1:10) in PBS and then 1 µL of diluted blood was added to 100 µL of staining buffer (PBS + 1% fetal bovine serum). Next, cells were incubated with (1:100) mouse anti-blood group H ab antigen-antibody (97-I) (from Abcam), (1:10) mouse anti-blood group H n/ab antigen-antibody (86-M) (from Creative Biolabs), or with (1:10) mouse IgM Isotype control (B11/7) (from Abcam) for 30 min on ice. Then, cells were washed twice with staining buffer and incubated with (1:100) PE goat anti-mouse IgM antibody from Invitrogen for 30 min at RT. For the staining with lectins, cells were incubated with (1:1000) FITC-conjugated anti-H lectin (L32476) for 15 min at RT. Then, cells were washed twice with staining buffer and incubated with BV421 mouse anti-CD235a (GA-R2) from BD Bioscience for 30 min on ice. Lastly, samples were washed once and resuspended in a staining buffer, and acquired in a MACSQuant Analyzer 10 flow cytometer (Miltenyi Biotec). Flow cytometry data were analyzed using FlowJo^TM^ v10.4. The determination of the population positive for antigen H was based on the isotype control.

### Reporting summary

Further information on research design is available in the Nature Portfolio Reporting Summary linked to this article.

## Supplementary information

Supplementary Information

Reporting Summary

## Data Availability

The atomic coordinates and structure factors have been deposited with the Protein Data Bank, access codes 7ZNZ (FucOBWT) and 7ZO0 (FucOBE541A). Previously published PDB structures used in this study are available under the accession codes: 2EAB, 7KMQ, 2RDY, 2EAD, 2EAE, 2EAC, and 4UFC. All other data are available from the corresponding authors on request. Source data are provided with this paper.

## Source data

Source Data
