# Supplementary material for: Turning universal O into rare Bombay type blood
Source: Nat Commun. 2023 Mar 30;14:1765. doi: 10.1038/s41467-023-37324-z (PMC10063614; doi:10.1038/s41467-023-37324-z)
Supplement: Supplementary file 1 — Supplementary Information [file 41467_2023_37324_MOESM1_ESM.pdf]

SUPPLEMENTARY INFORMATION

Itxaso Anso,[1,2,#] Andreas Naegeli,[3,#] Javier O. Cifuente,[1,2,#] Ane Orrantia,[4] Erica Andersson,[3] Olatz Zenarruzabeitia,[4] Alicia Moraleda-Montoya,[1] Mikel García-Alija,[1,2] Francisco Corzana,[5] Rafael A. Del Orbe,[6] Francisco Borrego,[4,7] Beatriz Trastoy,[1,2,7*] Jonathan Sjögren,[3,*] Marcelo E. Guerin.[1,2,7,*]

[1] Structural Glycobiology Laboratory, Biocruces Bizkaia Health Research Institute, Cruces University Hospital, 48903 Barakaldo, Bizkaia, Spain.
[2] Structural Glycobiology Laboratory, Center for Cooperative Research in Biosciences (CIC bioGUNE), Basque Research and Technology Alliance (BRTA), Bizkaia Technology Park, Building 801A, 48160 Derio, Spain.
[3] Genovis AB, Box 790, 22007 Lund, Sweden.
[4] Immunopathology Group, Biocruces Bizkaia Health Research Institute, Cruces University Hospital, 48903 Barakaldo, Bizkaia, Spain.
[5] Departamento Química and Centro de Investigación en Síntesis Química, Universidad de La Rioja, 26006 Logroño, Spain.
[6] Hematology and Hemotherapy Service, Cruces University Hospital, Biocruces Bizkaia Health Research Institute, 48903 Barakaldo, Bizkaia, Spain.
[7] Ikerbasque, Basque Foundation for Science, 48009 Bilbao, Spain.

[#]These authors contributed equally: Anso I., Naegeli A., Cifuente J. O.

*To whom correspondence should be addressed: Beatriz Trastoy, Structural Glycobiology Laboratory, IIS-Biocruces Bizkaia Cruces Plaza, 48903 Barakaldo, Bizkaia, Spain, beatriz.trastoy@gmail.com; Jonathan Sjogren, Genovis AB, Box 790, 22007 Lund, Sweden, jonathan.sjogren@genovis.com; Marcelo E. Guerin, Structural Glycobiology Laboratory, IIS-Biocruces Bizkaia Cruces Plaza, 48903 Barakaldo, Bizkaia, Spain, mrcguerin@gmail.com.

Short title: *Converting universal O into rare Bombay type blood*

Keywords: blood types, Bombay blood type, rare blood types, glycoside hydrolases, fucosidase, enzyme specificity, carbohydrate active enzymes.

Table of Contents

1. Supplementary Tables

Supplementary Table 1: Summary of biochemically characterized GH95 family members.

Supplementary Table 2: X-ray data collection and refinement statistics.

Supplementary Table 3: Summary of constructs.

2. Supplementary Figures

Supplementary Figure 1: Genomic organization of *Amuc_1120* gene in *A. muciniphila* ATCC BAA-835 strain.

Supplementary Figure 2: Recombinant production of FucOB.

Supplementary Figure 3: Electron density maps of the refined FucOB X-ray crystal structures.

Supplementary Figure 4: The catalytic mechanism of FucOB.

Supplementary Figure 5: Structural homologues of FucOB.

Supplementary Figure 6: Structure-based sequence alignment of N-terminal FucOB with GH95 homologues.

Supplementary Figure 7: Structure-based sequence alignment of C-terminal FucOB with GH95 homologues.

Supplementary Figure 8: 0.5 µs molecular (MD) dynamics simulations of the enzyme FucOB in complex with H, A, or B type antigens.

Supplementary Figure 9: Recombinant production of single point mutants of FucOB.

Supplementary Figure 10: Structural comparison of FucOB with GH95 homologues I.

Supplementary Figure 11: Structural comparison of FucOB with GH95 homologues II.

Supplementary Figure 12: FucOB concentration dependent conversion of universal O into rare Bombay type blood.

Supplementary Figure 13: FucOB converts both O^+ and O^- into rare Bombay type blood.

Supplementary Figure 14: FucOB conversion of universal O into rare Bombay type blood typing by anti-H lectin agglutination assay.

Supplementary Figure 15: Viability and integrity of converted Bombay RBCs.

Supplementary Figure 16: Gating strategy for the analysis of antigen H expression in RBCs.

3. Supplementary References

1. Supplementary Tables

Supplementary Table 1. Summary of biochemically characterized GH95 family members.

Protein Name	Bacterial Organism	Characterized activity	Substrates	PDB code	Reference
FucOB<br><br>Uniprot code: B2UR61	*Akkermansia muciniphila*	α-1,2-L-fucosidase	Type I, Type II, Type V(2'-fucosyllactose) H antigens	7ZNZ<br>7ZO0	This work
XacAfc95<br><br>Uniprot code: Q8PLM1	*Xanthomonas citri*	α-L-fucosidase	pNP-α-L-fucopyranoside	7KMQ	Vieira et al. *Nat. Commns.*12: 4049 (2021).[1]
*Bb*AfcA<br><br>Uniprot code: Q6JV24	*Bifidobacterium bifidum*	α-1,2-L-fucosidase	Type II, Type V (2'-fucosyl-lactose)<br><br>H antigens<br>Lacto-*N*-fucopentaose I	2EAB<br>2EAC<br>2EAD<br>2EAE	Katayama et al. *J. Bacteriol.* 186:4885–4893 (2004).[2] Nagae et al. *J. Biol. Chem.* 282, 18497–18509 (2007).[3]
BuGH95; BACUNI_00326<br><br>Uniprot code: A7UYF5	*Bacteroides uniformis*	α-L-fucosidase	2-chloro-4-nitrophenyl α-l-fucoside (Fuc-α-CNP)<br><br>2′-fucosyllactose (Type V H antigen)<br><br>3′-fucosyllactose		Déjean et al. *Appl. Environ. Microbiol.;*85:e 01491–19 (2019).[4]
BfGH95; HMPREF9446_01800<br><br>Uniprot code:F3PST8	*Bacteroides fluxus*	α-L-fucosidase	2-chloro-4-nitrophenyl α-l-fucoside (Fuc-α-CNP)<br><br>2′-fucosyllactose (Type V H antigen)<br><br>3′-fucosyllactose		Déjean et al. *Appl. Environ. Microbiol.;*85:e 01491–19 (2019). [4]
DgGH95; HMPREF9455_00301<br><br>Uniprot code F5IT84	*Dysgonomonas gadei*	α-L-fucosidase	2-chloro-4-nitrophenyl α-l-fucoside (Fuc-α-CNP)<br><br>2′-fucosyllactose (Type V H antigen)<br><br>3′-fucosyllactose<br><br>Fucosyl-α(1,6)-*N*-acetylglucosamine		Déjean et al. *Appl. Environ. Microbiol.;*85:e 01491–19 (2019). [4]
Blon_2335<br><br>Uniprot code:B7GNN7	*Bifidobacterium longum subsp. infantis*	α-L-fucosidase	2-chloro-4-nitrophenyl α-l-fucoside (Fuc-α-CNP<br><br>2′-fucosyllactose (Type V H antigen)<br><br>3′-fucosyllactose<br><br>Fuc α-l,2-Gal H antigen disaccharide<br><br>HMO (Human Milk Oligosaccharides)		Sela et al. *Appl. Environ. Microbiol.* 78:795–803 (2012).[5]
CjAfc95A<br><br>Uniprot code: B3PBE3	*Cellvibrio japonicus*	α-L-fucosidase	2-chloro-4-nitrophenyl α-l-fucoside (Fuc-α-CNP		Larsbrink et al. *Mol. Microbiol.* 94:418–433 (2014).[6]

Afc3; CPF_2129<br><br>Uniprot code: A0A0H2YQB3	*Clostridium perfringens*	α-1,2-L-fucosidase	Fuc α-l,2-Gal H antigen disaccharide<br><br>Porcine gastric mucin (PGM)		Fan et al. *J. Basic Microbiol.* 56:347–357 (2016).[7]
RiFuc95<br><br>Uniprot code: C0FT20	*Roseburia inulinivorans*	α-1,2-L-fucosidase	Type I H antigen		Pichler *et al. Nat Commun.* 11:3285 (2020).[8]
RUMGNA_00842<br><br>Uniprot code: A7AZW8	*Ruminococcus gnavus*	α-L-fucosidase	2′-fucosyllactose (Type V H antigen)<br><br>3′-fucosyllactose		Wu et al. *Cell Mol. Life Sci.* 78:675–693 (2021).[9]
SpGH95; SP_1654<br><br>Uniprot code: A0A0H2UR14	*Streptococcus pneumoniae*	α-1,2-L-fucosidase	Type I, II, IV, V H antigen<br><br>Lewis^B tetrasaccharide<br><br>Lewis^y tetrasaccharide		Hobbs et al. *J Biol. Chem.* 294:12670–12682 (2019).[10]
CsFase I<br><br>WP_047034338	*Elizabethkingia meningoseptica*	α-1,2-fucosidase	pNP-α-L-fucopyranoside<br><br>Type I, II, IV, V H antigen<br><br>Fuc α-l,2-Gal H antigen disaccharide		Tiansheng et al. bioRxiv doi:10.1101/695213 (2019).[11]
BACOVA_03438<br><br>Uniprot code: A7M011	*Bacteroides ovatus*	α-L-galactosidase	corn bran xylan (CX)	4UFC	Rogowski et al. *Nat Commun.* 6:7481 (2015).[12]
BT1010<br><br>Uniprot code: Q8A907	*Bacteroides thetaiotaomicron*	α-L-galactosidase	rhamnogalacturonan-II (RGII)		Ndeh et al. Nature 544:65–70 (2017).[13]

*Three eukaryotic α-1,2-L-fucosidases were additionally reported: (i) Fuc95A from *Arabidopsis thaliana* (Uniprot code Q8L7W8;),[14] (ii) α-1,2-L-fucosidase AN8149.2 from *Aspergillus nidulans* FGSC A4 (Uniprot codes C8V6V7 and Q5AU81)[15] and α-1,2-L-fucosidase LlFuc from *Lilium longiflorum* (Uniprot code A8J657).[16]

Supplementary Table 2. X-ray data collection and refinement statistics.

	FucOB	FucOB$_{E541A}$
PDB code	7ZNZ	7ZO0
Beamline	X06DA-PXIII (SLS)	BL13-XALOC
Wavelength	0.9792	0.97926
Resolution range	47.33 - 1.8 (1.864 - 1.8)	52.24 - 1.95 (2.02 - 1.95)
Space group	P 21 21 21	P 1 21 1
Unit cell	53.67 100.40 156.98 90 90 90	53.58 87.50 95.58 90 102 90
Total reflections	975882 (65783)	423928 (43184)
Unique reflections	79452 (7833)	62656 (6188)
Multiplicity	12.3 (8.4)	6.8 (7.0)
Completeness (%)	99.93 (99.80)	99.84 (99.77)
Mean I/sigma(I)	26.40 (1.84)	12.84 (1.91)
Wilson B-factor	31.84	27.38
R-merge	0.05474 (1.058)	0.1299 (1.223)
R-meas	0.05709 (1.126)	0.1409 (1.323)
R-pim	0.01601 (0.3804)	0.05409 (0.4985)
CC1/2	1 (0.813)	0.998 (0.648)
CC*	1 (0.947)	0.999 (0.887)
Reflections used in refinement	79417 (7830)	62627 (6188)
Reflections used for R-free	3972 (392)	2951 (295)
R-work	0.1899 (0.3396)	0.2033 (0.2901)
R-free	0.2177 (0.3568)	0.2142 (0.3208)
CC(work)	0.954 (0.875)	0.957 (0.744)
CC(free)	0.899 (0.861)	0.948 (0.708)
Number of non-hydrogen atoms	6317	6012
macromolecules	5871	5730
ligands	56	30
Protein residues	422	761
RMS(bonds)	0.012	0.004
RMS(angles)	1.18	0.62
Ramachandran favored (%)	96.71	96.97
Ramachandran allowed (%)	3.16	2.90
Ramachandran outliers (%)	0.13	0.13
Rotamer outliers (%)	0.84	0.89
Clashscore	4.06	3.94
Average B-factor	43.18	29.86
macromolecules	42.99	29.55
ligands	50.81	40.02
solvent	45.41	35.69

Statistics for the highest-resolution shell are shown in parentheses

Supplementary Table 3. Summary of constructs.

Description	Template	Primers (5' to 3')
FucOB_W378A	pET29a-*fucOB*	1) GTCAAACCGCCGGCGGCCTGCGACTACC<br>2) GGTAGTCGCAGGCCGCCGGCGGTTTGAC
FucOB_H383A	pET29a-*fucOB*	1) GTGGGCCTGCGACTACGCTAACAACATCAACGTCC<br>2) GGACGTTGATGTTGTTAGCGTAGTCGCAGGCCCAC
FucOB_N385A	pET29a-*fucOB*	1) CTGCGACTACCATAACGCCATCAACGTCCAGATGG<br>2) CCATCTGGACGTTGATGGCGTTATGGTAGTCGCAG
FucOB_N387A	pET29a-*fucOB*	1) CTACCATAACAACATCGCCGTCCAGATGGCGTATTGG<br>2) CCAATACGCCATCTGGACGGCGATGTTGTTATGGTAG
FucOB_T443A	pET29a-*fucOB*	1) GGACGGTGCGCGCCTCCCAGAATATCTTC<br>2) GAAGATATTCTGGGAGGCGCGCACCGTCC
FucOB_S444A	pET29a-*fucOB*	1) GACGGTGCGCACCGCCCAGAATATCTTC<br>2) GAAGATATTCTGGGCGGTGCGCACCGTC
FucOB_W453A	pET29a-*fucOB*	1) CTTCGGTGGCAACGGCGCGCAGTGGAACATTC<br>2) GAATGTTCCACTGCGCGCCGTTGCCACCGAAG
FucOB_H613A	pET29a-*fucOB*	1) GACGGACCACCGCGCTACGTCCCACCTTTTTG<br>2) CAAAAAGGTGGGACGTAGCGCGGTGGTCCGTC
FucOB_W655A	pET29a-*fucOB*	1) GCCGCCGTTCCGCGACGTGGCCGTG<br>2) CACGGCCACGTCGCGGAACGGCGGC
FucOB_H693A	pET29a-*fucOB*	1) CGAATATGCTGACTACTGCTCCCCCTATGCAGATGG<br>2) CCATCTGCATAGGGGGAGCAGTAGTCAGCATATTCG
FucOB_D699A	pET29a-*fucOB*	1) CTATGCAGATGGCCGGCAACTTCGGCATTG<br>2) CAATGCCGAAGTTGCCGGCCATCTGCATAG

2. Supplementary Figures

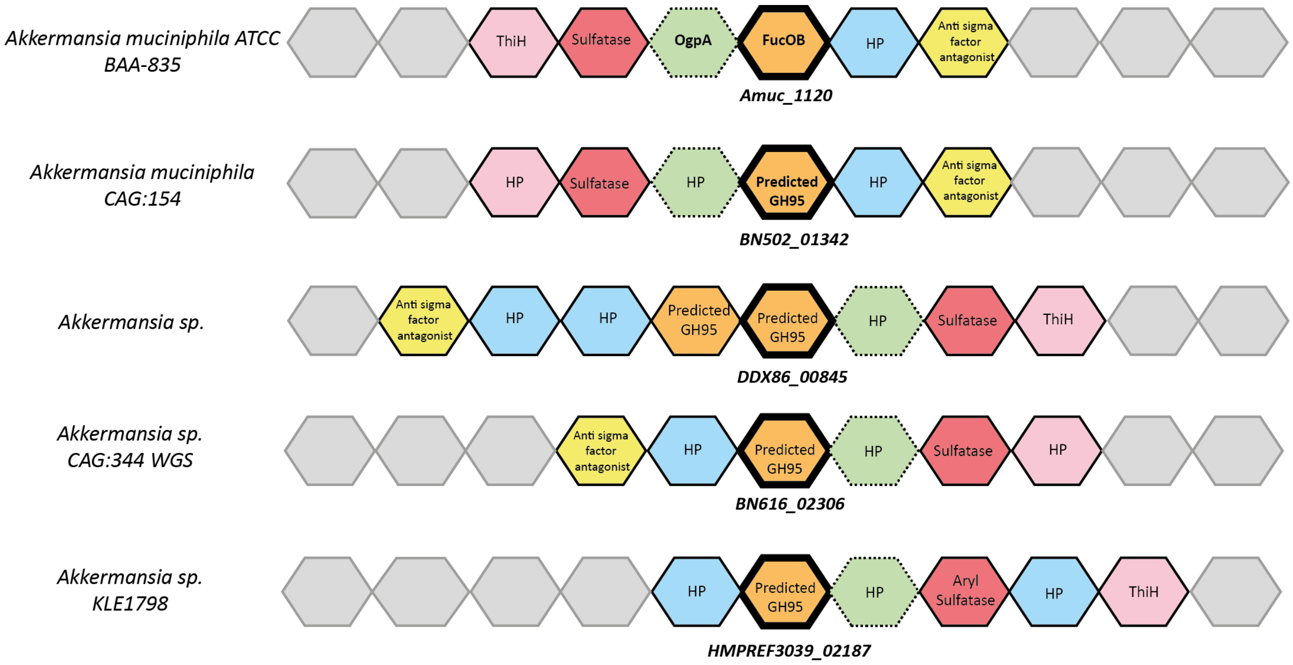

Supplementary Fig. 1 | Genomic organization of *Amuc_1120* gene in *A. muciniphila ATCC BAA-835* strain. Linear distribution of similar functionality genes represented with hexagons upstream and downstream from *fucOB* gene (*Amuc_1120)* in *A. muciniphila ATCC BAA-835 strain* in the top row and *FucOB* gene homologues in *CAG:154, sp. (DDX86_00850), CAG:344 WGS (BN616_02307)* and *KLE1798 (HMPREF3039_02188)* strains respectively in the following rows according to the National Center for Biotechnology Information (NCBI) Gene and Genome databases (https://www.ncbi.nlm.nih.gov). Similar functionality genes are highlighted in the same colors. The *fucOB* gene and its homologues are highlighted in orange in the middle of the linear genomic representation. HP, hypothetical protein, refers to genes predicted to encode an unknown function protein, represented in blue. In light green dashed line boxes indicates the *ogpA* gene and its homologues, sulfatases in red, anti-sigma factor encoding genes in yellow and a dihydroxy-acid dehydratase related to thiamin and biotin synthesis encoding genes in light pink. Grey hexagons represent those non-conserved genes along the different *Akkermansia* species.

a

MHHHHHHENLYFQGSGADKPSASNLIWSDEPAVVVYPQEDKNSEGSFGKYRKPASVWEAE-
GYPIGNGRVGAMIFSAPGRERLALNEISLWSGGANPGGGYGYGPDAGTNQFGNYLPFGDLFVDFKKG
DQPASLSVEDFTRSLDLRDGIHKVNYKADGVTYDREAFSSTPANVLVLNYKASKPGQFSADFSVNSQ
LGADISAKGSVITWKGMLKNGMNYEGRVLIRPKGGTLSASGDKISVKNADSCMVVIAMETDYLMDYK
KDWKGESPSRKLDRYAAKAASADYAALKQAHISQYKSMFDRVKVNFGKTEEDVAKLPTPKRLEAYKK
NPADPDLEETMFQFGRYLLLSSSRPGTLPANLQGLWNDYVKPPWACDYHNNINVQMAYWGAEPANLS
ECHEALVNYVEAMAPGCRDASQANKGFNTKDGKPVRGWTVRTSQNIFGGNGWQWNIPGAAWYALHIW
EHYAFTGDRKYLEKQAYPLMKEICHFWEDHLKELGAGGEGFKTNGKDPSEEEKKDLADVKAGTLVAP
NGWSPEHGPREDGVMHDQQLIAELFSNTIKAARILGKDAAWAKSLEGKLKRLAGNKIGKEGNLQEWM
IDRIPKTDHRHTSHLFAVFPGNQISKLKTPKLAEAARLSLEWRGTTGDSRRSWTWPWRTALWARLGE
GNKAHEMVQGLLKFNTLPNMLTTHPPMQMDGNFGIVGGICEMLVQSHAGGLDIMPSPVEAWPEGSVK
GLKARGNVTVDFSWKDGKVSNVKLYSAQPKVLPVRVNGKMTRMKTLPLKSGAGSSQPAAR

b

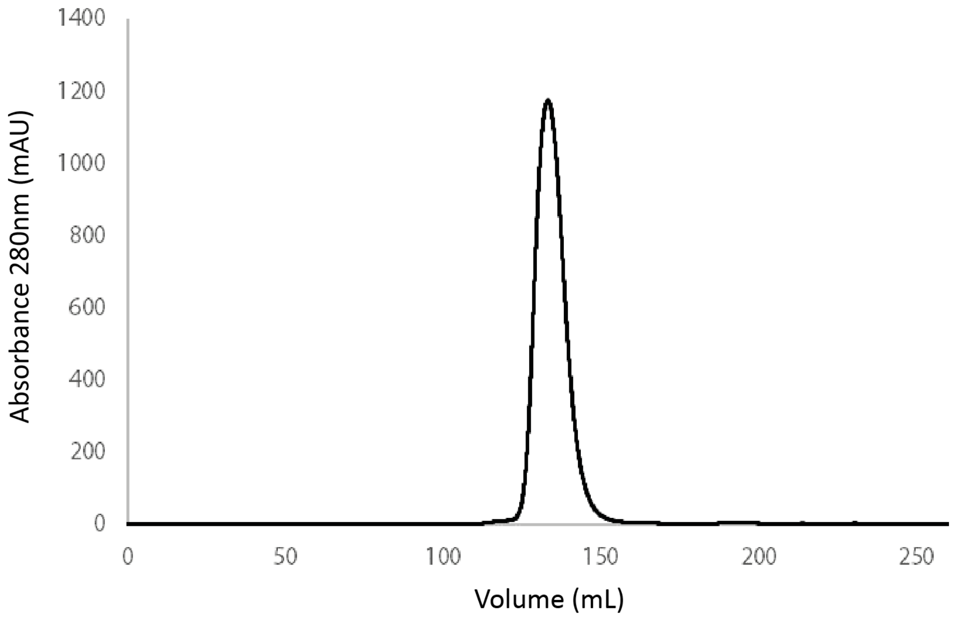

c

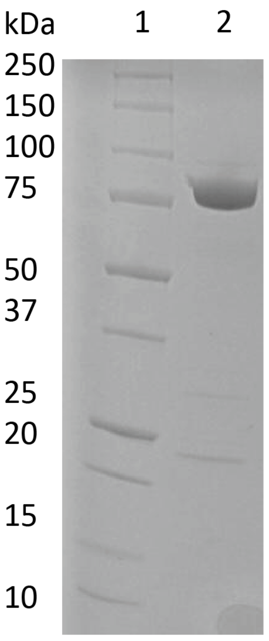

Supplementary Fig. 2 | Recombinant production of FucOB. a Schematic representation of FucOB sequence. The catalytic residues are colored in green. N-terminal his-tag, TEV protease recognition site and GSGA linker sequences are colored in orange. **b** Pre-crystallization SEC purification. **c** SDS-PAGE analysis of FucOB. This profile is always repeated in the performed three different purifications. Source data are provided as a Source Data file.

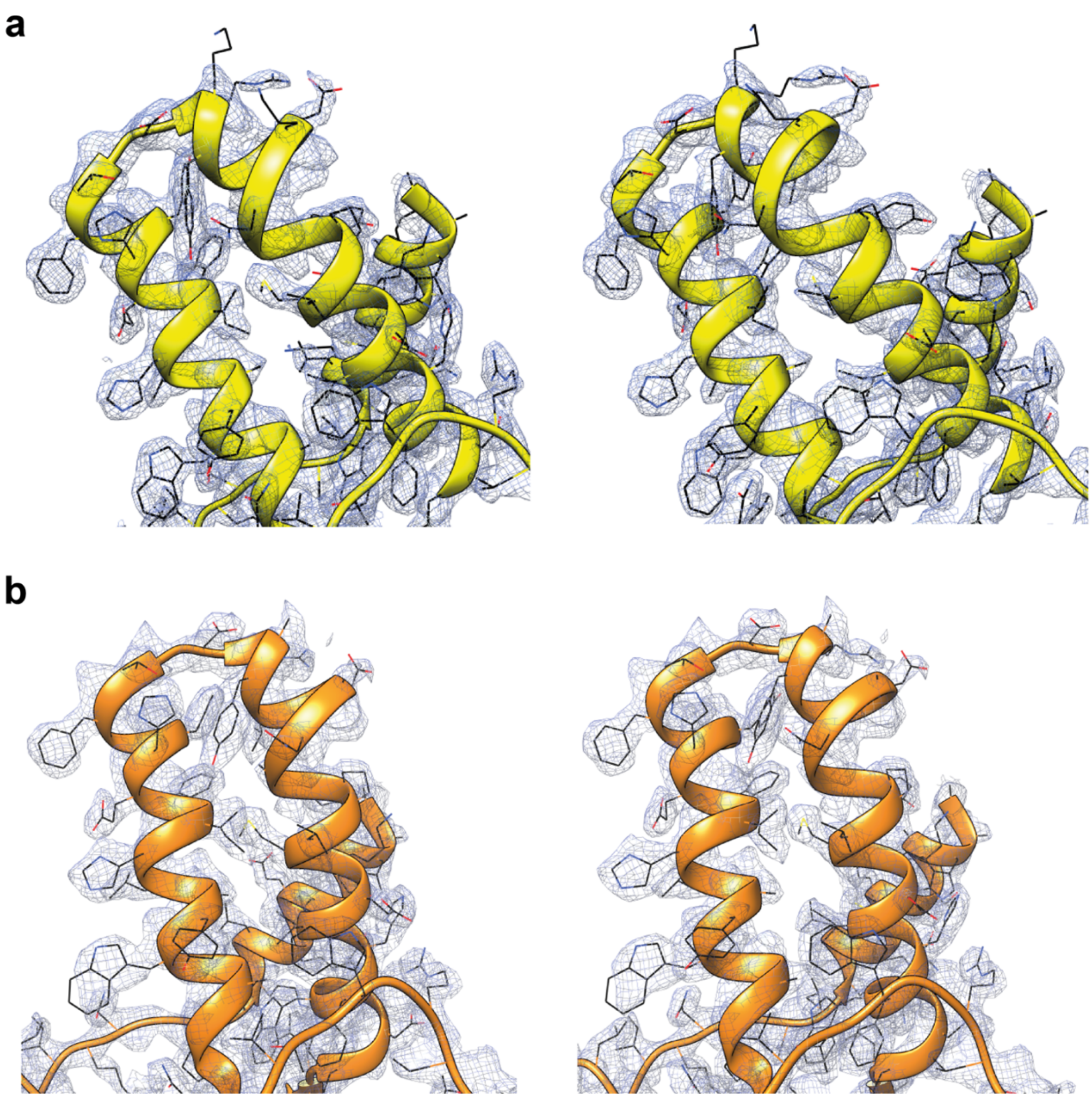

Supplementary Fig. 3 | Electron density maps of the refined FucOB X-ray crystal structures. Final electron density maps (2mFo-DFc contoured at 1σ) corresponding to unliganded FucOB (**a**) and FucOB$_{E541A}$ (**b**). Cross-eyed stereo figures are shown.

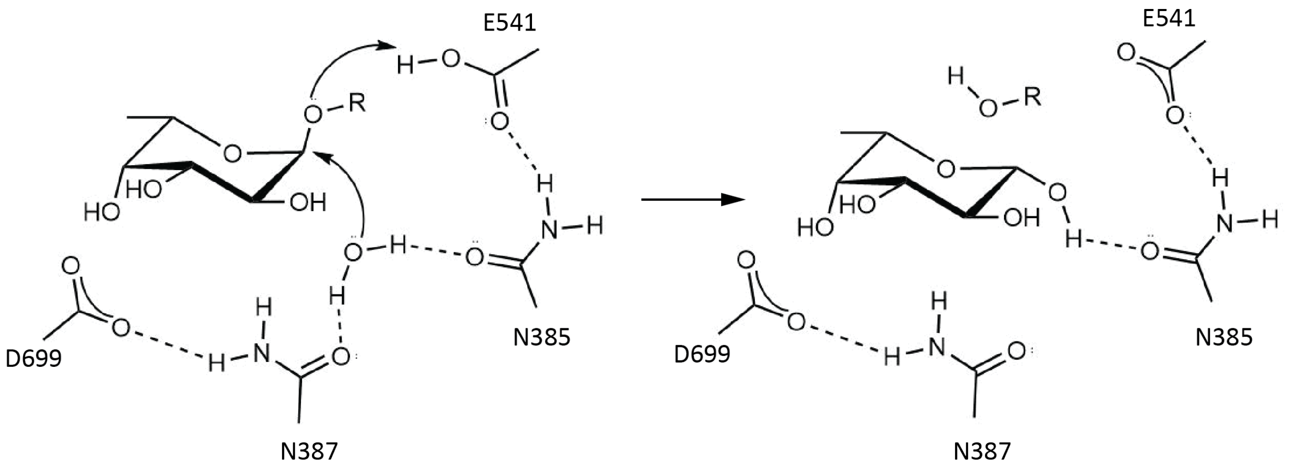

R: Common precursor

Supplementary Fig. 4 | The catalytic mechanism of FucOB. FucOB follows a single displacement inverting catalytic mechanism, as proposed for *Bb*AfcA and other GH95 family members. A conserved glutamic acid (E541) acts as a general acid catalyst. A water molecule acts as a general base in the reaction, activated by two asparagine residues (N385 and N387) and an aspartic acid residue (D699) to perform the nucleophilic attack to the fucose.

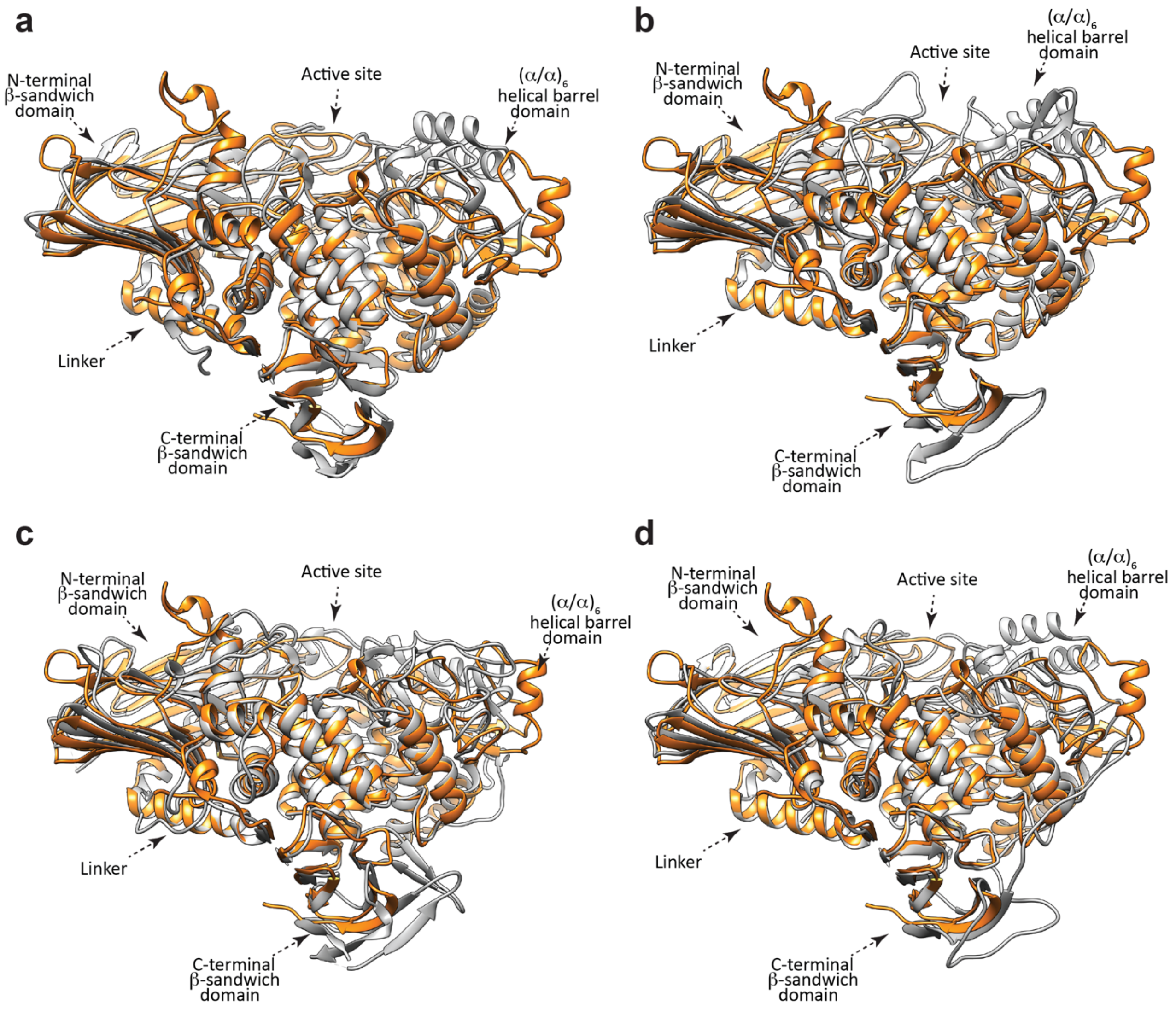

Supplementary Fig. 5 | Structural homologues of FucOB. Structural superposition of the X-ray crystal structure of FucOB and structural homologues (grey): **a** α-1,2-fucosidase *Xac*Afc95 from *Xanthomonas citri* (PDB code 7KMQ), **b** a putative GH95 member from *Bacillus halodurans* (PDB code 2RDY), **c** α-1,2-fucosidase *Bb*AfcA from *Bifidobacterium bifidum* (PDB code 2EAB), **d** α-L-galactosidase BACOVA_03438 from *Bacteroides ovatus* (PDB code 4UFC).

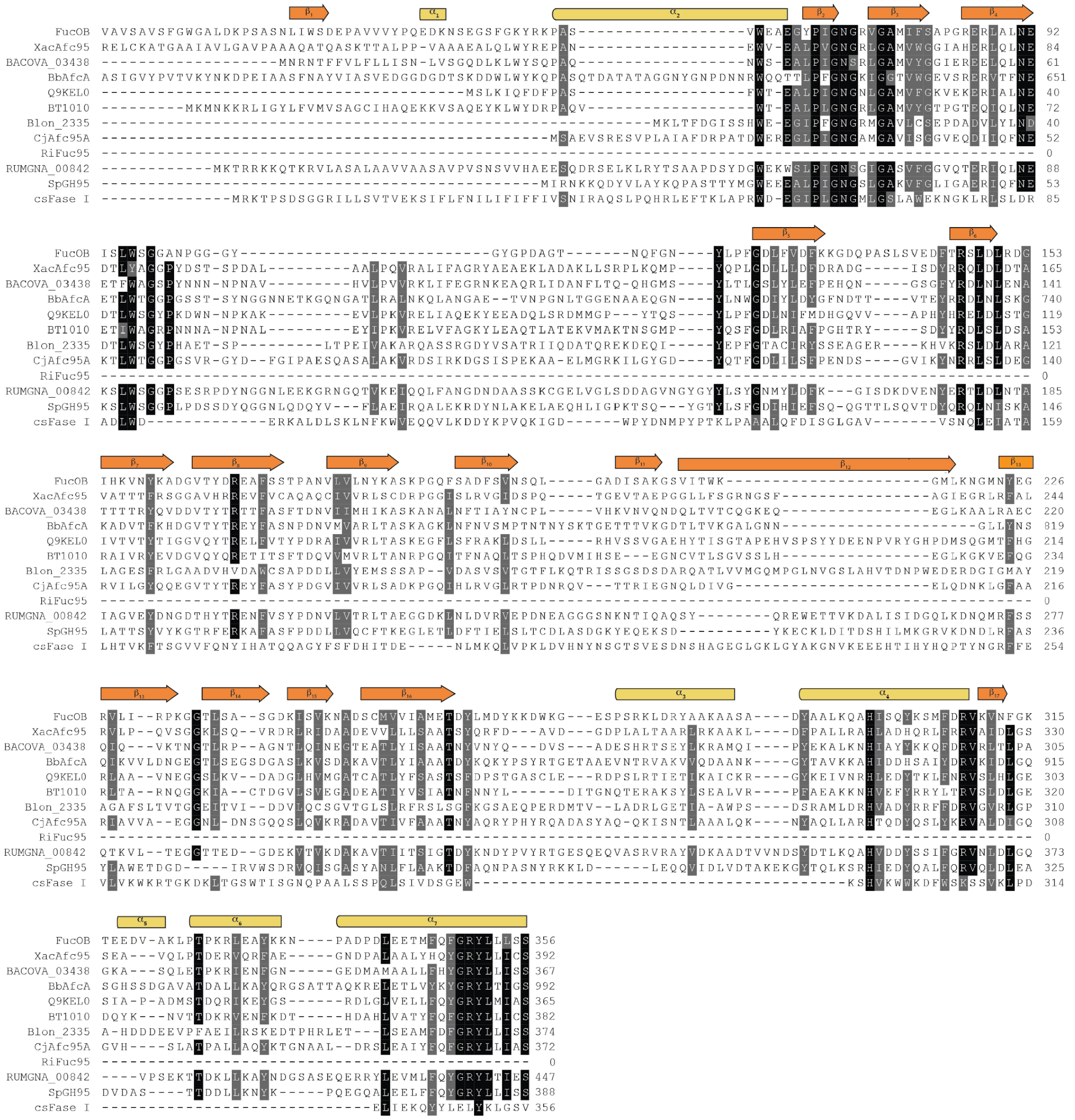

Supplementary Fig. 6 | Sequence alignment of N-terminal FucOB with GH95 homologues. Comparison of FucOB from *A. muciniphila* and homologues from GH95 family, according to CAZY database; XacAfc95 from *Xanthomonas citri* (Q8PLM1, Uniprot Code), BACOVA_034338 from *Bacteroides ovatus (*A7M011, Uniprot Code), BbAfcA from *Bifidobacterium bifidum (*Q6JV24, Uniprot Code), a putative GH family protein from *Bacillus halodurans* (Q9KEL0, Uniprot code), BT1010 from *Bacteroides thetaiotaomicron (*Q8A907, Uniprot code), Blon_2335 from *B. longum (*B7GNN7, Uniprot code), CjAfc95A from *Cellvibrio japonicas* (B3PBE3, Uniprot code), RiFuc95 from *Roseburia inulinivorans* (C0FT20, Uniprot code), RUMGNA_00842 from *R. gnavus* (A7AZW8, Uniprot code), SpGH95 from *Streptococcus pneumonia* (A0A0H2UR14, Uniprot code) and csFASE I from *Elizabethkingia meningoseptica* (WP_047034338 accession number). The distribution of secondary structure elements of FucOB is displayed above the alignment.

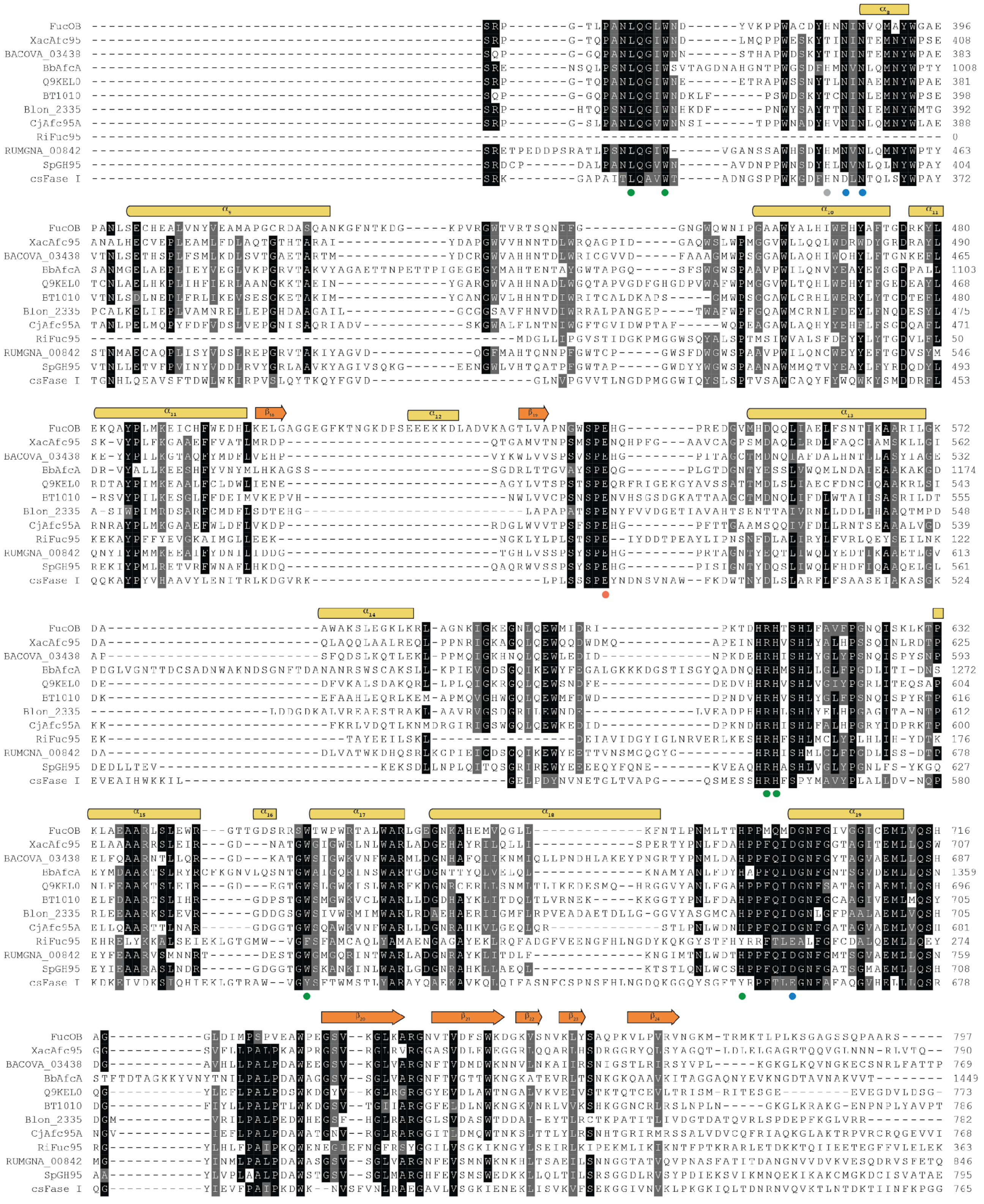

Supplementary Fig. 7 | Sequence alignment of C-terminal FucOB with GH95 homologues. Comparison of FucOB from *A. muciniphila* and homologues from GH95 family, according to CAZY database; XacAfc95 from *Xanthomonas citri* (Q8PLM1, Uniprot Code), BACOVA_034338 from *Bacteroides ovatus (*A7M011, Uniprot Code), BbAfcA from *Bifidobacterium bifidum (*Q6JV24, Uniprot Code), a putative GH family protein from *Bacillus halodurans* (Q9KEL0, Uniprot code), BT1010 from *Bacteroides thetaiotaomicron (*Q8A907, Uniprot code), Blon_2335 from *B. longum (*B7GNN7, Uniprot code), CjAfc95A from *Cellvibrio japonicas* (B3PBE3, Uniprot code), RiFuc95 from *Roseburia inulinivorans* (C0FT20, Uniprot code), RUMGNA_00842 from *R. gnavus* (A7AZW8, Uniprot), SpGH95 from *Streptococcus pneumonia* (A0A0H2UR14, Uniprot code) and

csFASE I from *Elizabethkingia meningoseptica* (WP_047034338 accession number). The distribution of secondary structure elements of FucOB is displayed above the alignment. Catalytic residues are highlighted with orange dots. Conserved residues in binding site are highlighted with green dots. Non-conserved residue in binding site is highlighted in grey dot.

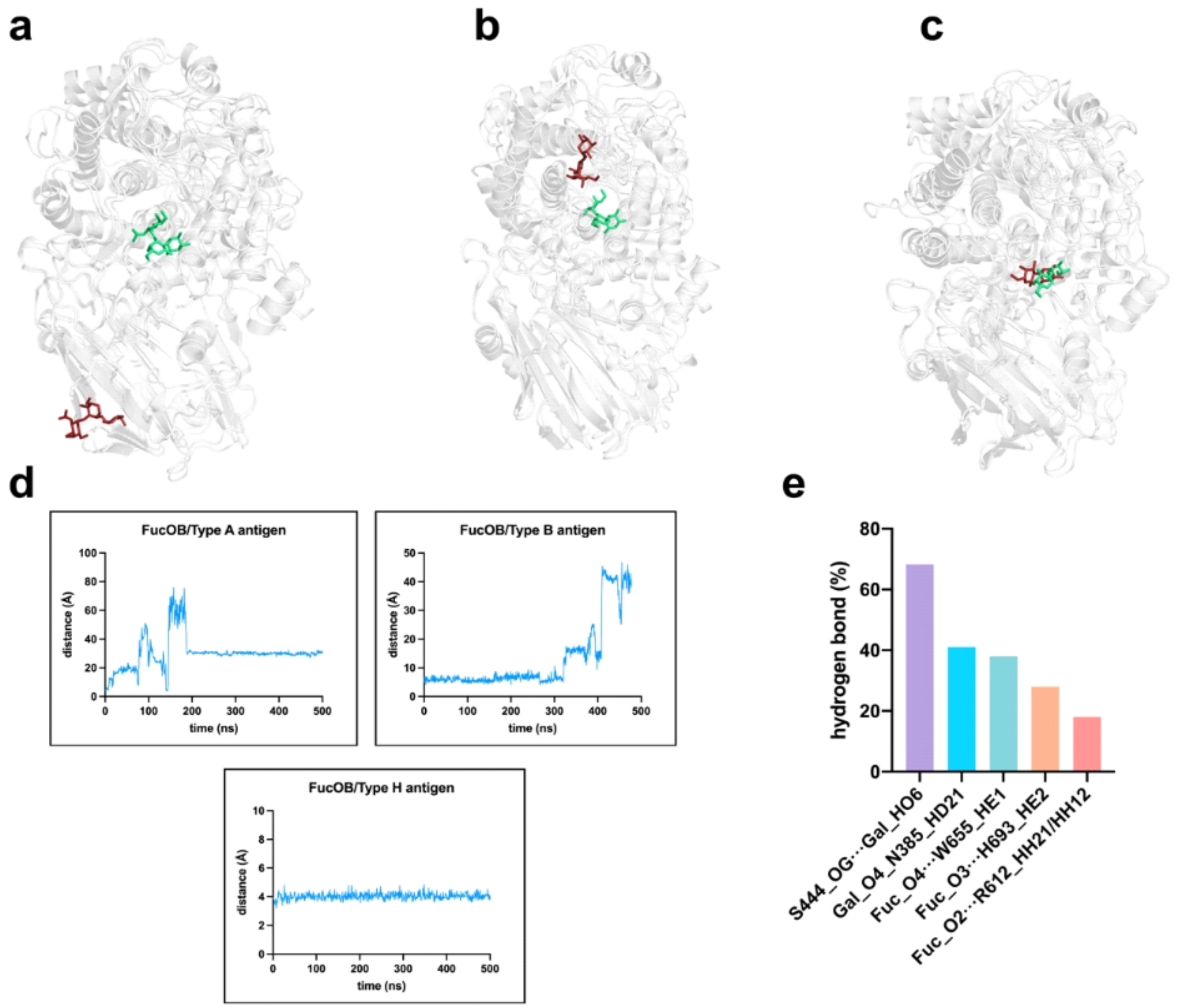

Supplementary Fig. 8 | 0.5 µs molecular dynamics (MD) simulations of the enzyme FucOB complexes with H, A or B type antigens. Initial (in green) and final (in red) structure of A (**a**), B (**b**) and H (**c**) antigens in complex with FucOB (shown as white ribbons). **b** Monitoring distance between the center of the aromatic ring of W378 and the methyl group of the fucose residue during the MD simulation of the FucOB complexed to H (**a**), A (**b**) and B (**c**) antigens. **e** Hydrogen bonds made between FucOB and H antigen derived from the MD simulations.

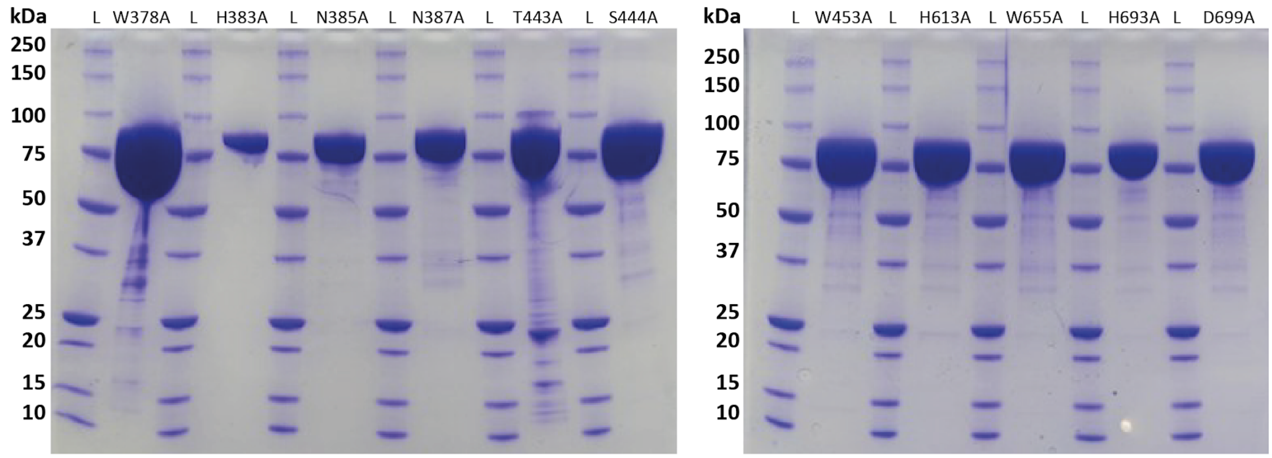

Supplementary Fig. 9 | Recombinant production of single point mutants of FucOB. SDS-PAGE analysis of the purified single point mutants of FucOB: W378A, H383A, N385A, N387A, T443A, S444A, W453A, H613A, W655A, H693A and D699A. The protein ladder is shown as L. This SDS-PAGE profile was obtained from single mutants of FucOB purification. Source data are provided as a Source Data file.

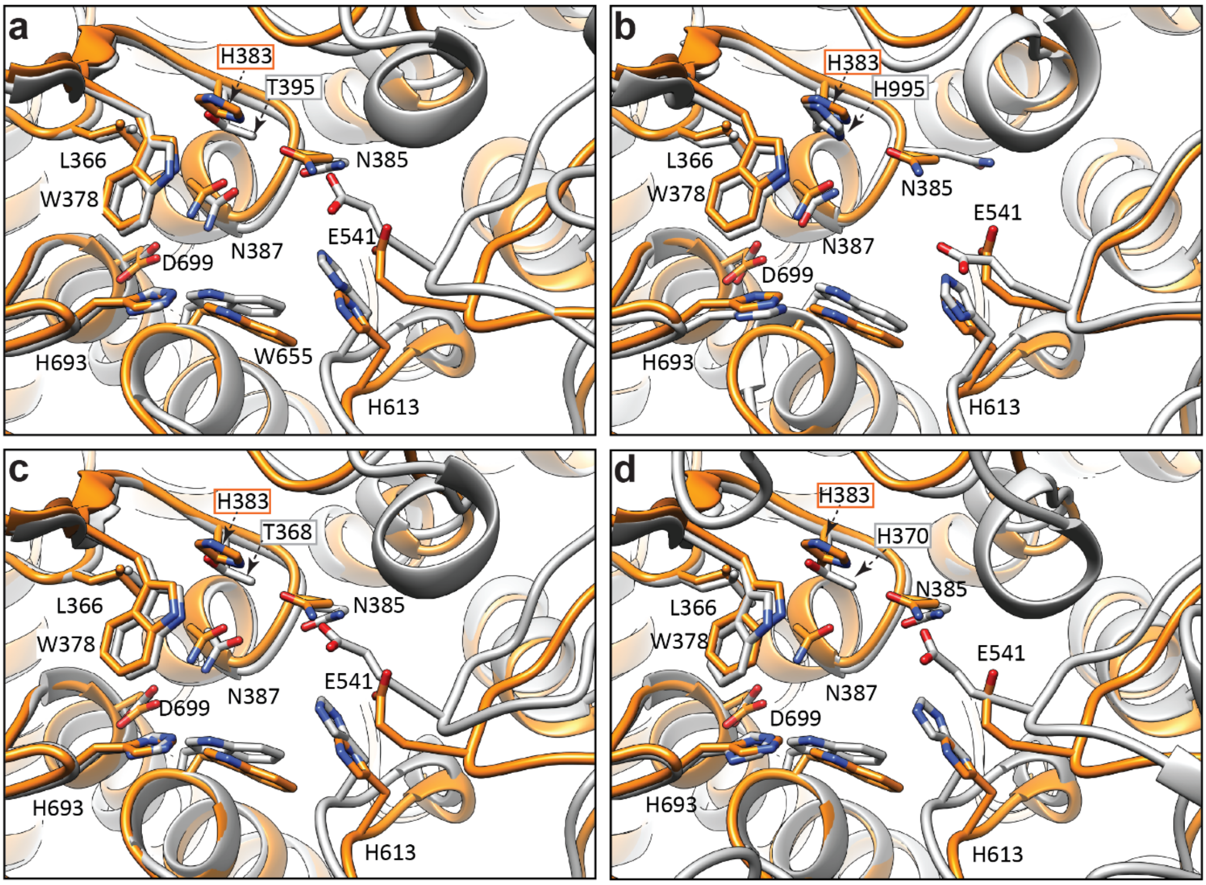

Supplementary Fig. 10 | Structural comparison of FucOB with GH95 homologues I. Binding site comparison between FucOB (in orange) and crystal structures of GH95 homologues (in grey). It is worth mentioning that the active site's architecture along the GH95 family is highly conserved. FucOB conserved residues that participate in binding are highlighted. The non-conserved residue is squared in orange (FucOB) or grey (homologues). **a** α-1,2-fucosidase *Xac*Afc95 from *Xanthomonas citri* (PDB code 7KMQ), **b** α-1,2-fucosidase *Bb*AfcA from *Bifidobacterium bifidum* (PDB code 2EAB), **c** a putative GH95 member from *Bacillus halodurans* (PDB code 2RDY) **d** α-L-galactosidase BACOVA_03438 from *Bacteroides ovatus* (PDB code 4UFC).

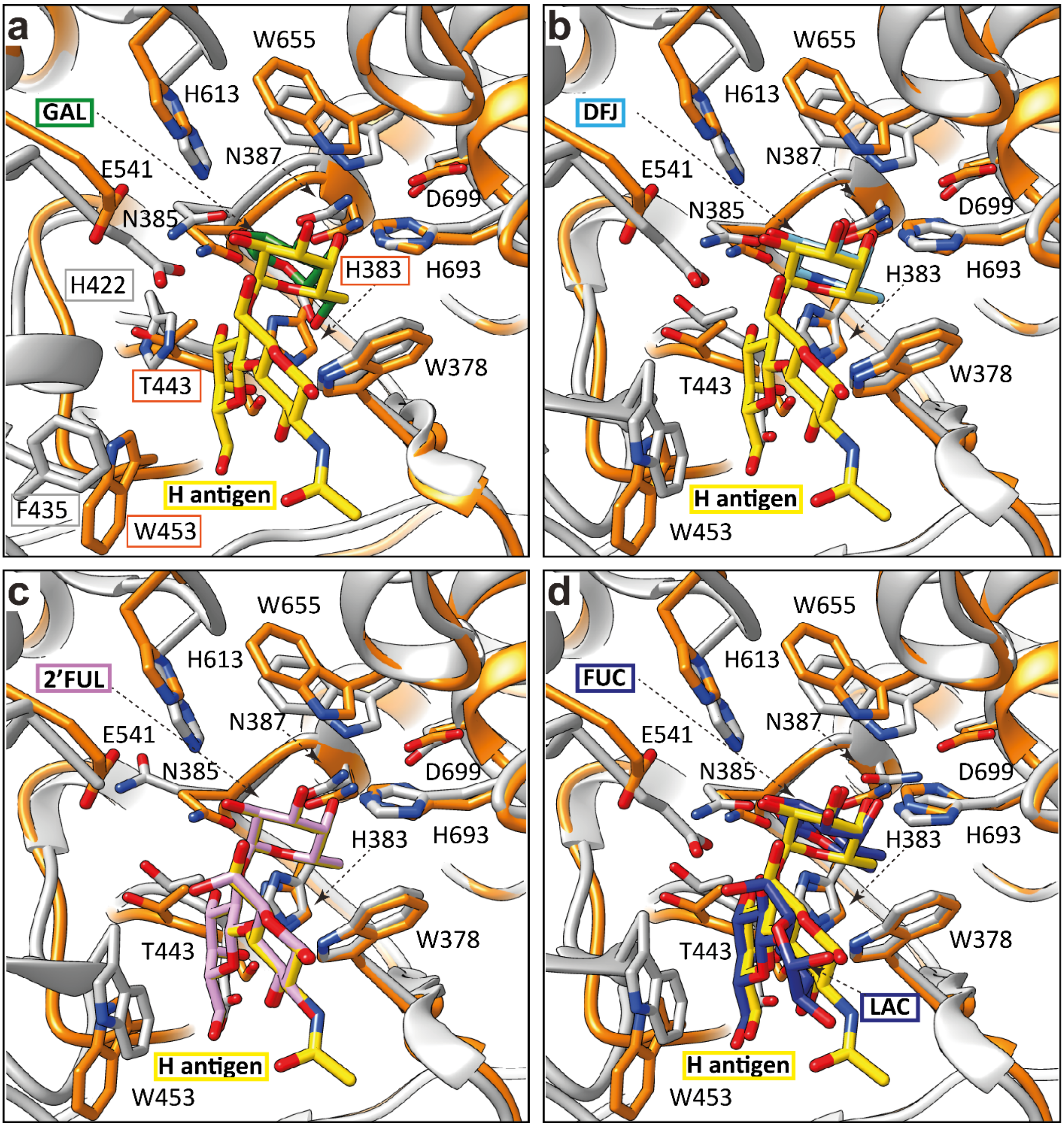

Supplementary Fig. 11 | Structural comparison of FucOB with GH95 homologues II. Binding site comparison between the FucOB (in orange) in complex with the H Type II antigen obtained by *in silico* molecular docking calculations (in yellow) with that of crystal structures of GH95 homologues in complex with ligands (in grey). FucOB conserved residues that participate in binding are highlighted. **a** α-L-galactosidase BACOVA_03438 from *Bacteroides ovatus* in complex with galactose (PDB code 4UFC; Gal residue in green), **b** α-1,2-fucosidase *Bb*AfcA from *Bifidobacterium bifidum* in complex with DFJ inhibitor (PDB code 2EAC; DFJ in light blue), **c** *Bb*AfcA from *Bifidobacterium bifidum* in complex with the substrate 2'FL (PDB code 2EAD; 2'FL in pink) **d** α-1,2-fucosidase *Bb*AfcA from *Bifidobacterium bifidum* in complex with the products α-L-Fuc and Galβ1–4Glc (PDB code 2EAC; Fuc and Lac in blue).

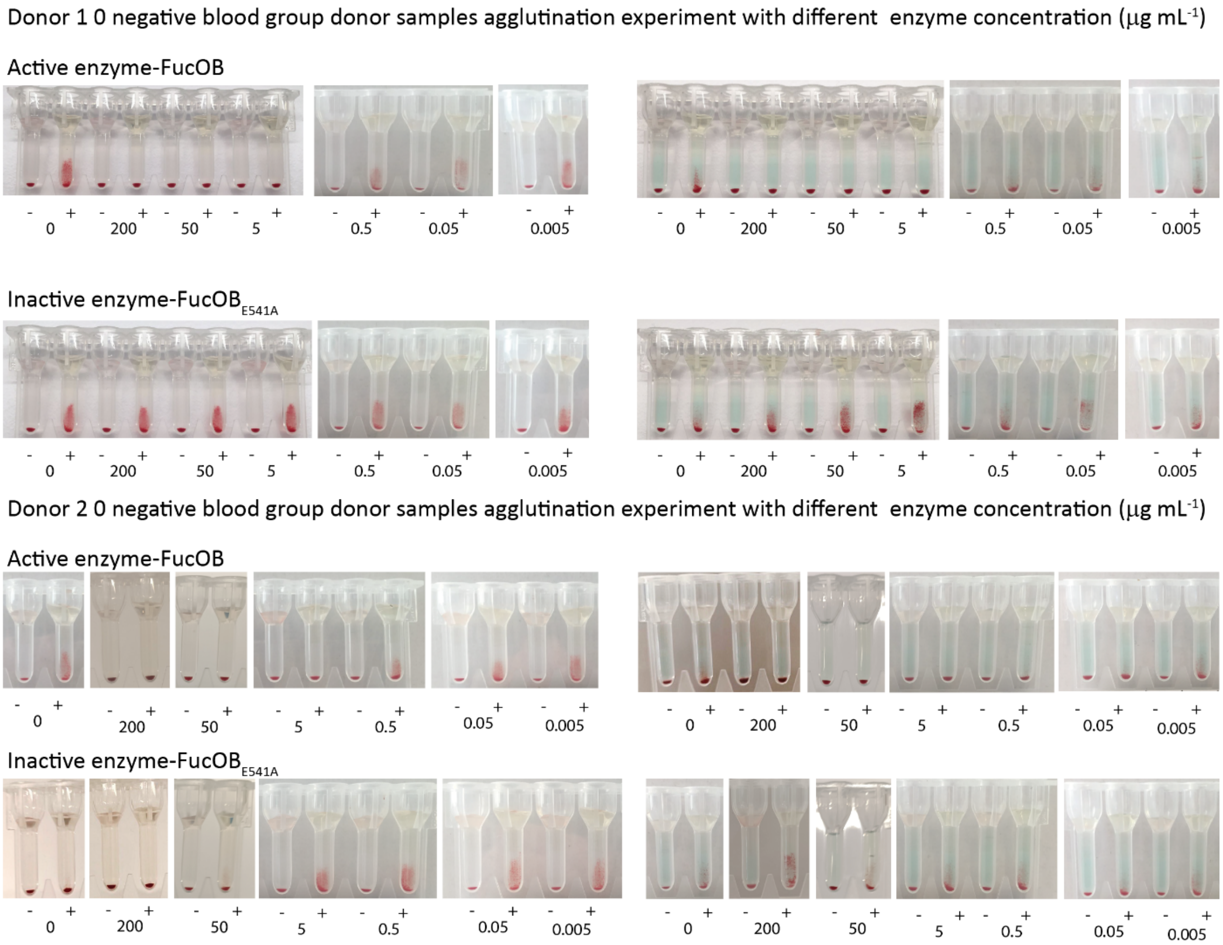

Supplementary Fig. 12 | FucOB concentration dependent conversion of universal O into rare Bombay type blood. Agglutination assay results from 2 different donor O blood group samples (donor 1 in first and second row, donor 2 in third and fourth row). In the left panels appear the pictures of the gel-column of DG Gel Neutral cards and in the right panels appear the pictures from DG Gel Coombs cards. 1 and 3 rows show samples enzymatically treated with different concentrations of active FucOB (0, 200, 50, 5, 0.5 0.05 and 0.005 μg mL^{-1}). 2 and 4 rows show samples enzymatically treated with different concentrations of inactive FucOB$_{E541A}$ (0, 200, 50, 5, 0.5 0.05 and 0.005 μg mL^{-1}). Each sample was incubated in the absence (-) and presence (+) of naturally containing anti-H antibodies Bombay serum. The pellet in the bottom of the gel column is a negative result, meaning that there is no agglutination or hemolysis in the sample. When clumps of cells appear throughout the gel column shows a positive result, meaning that cells are agglutinated in the sample. It is worth noting that each agglutination card has 8 buffered tubes to perform the experiments. Therefore, the results are shown in patches.

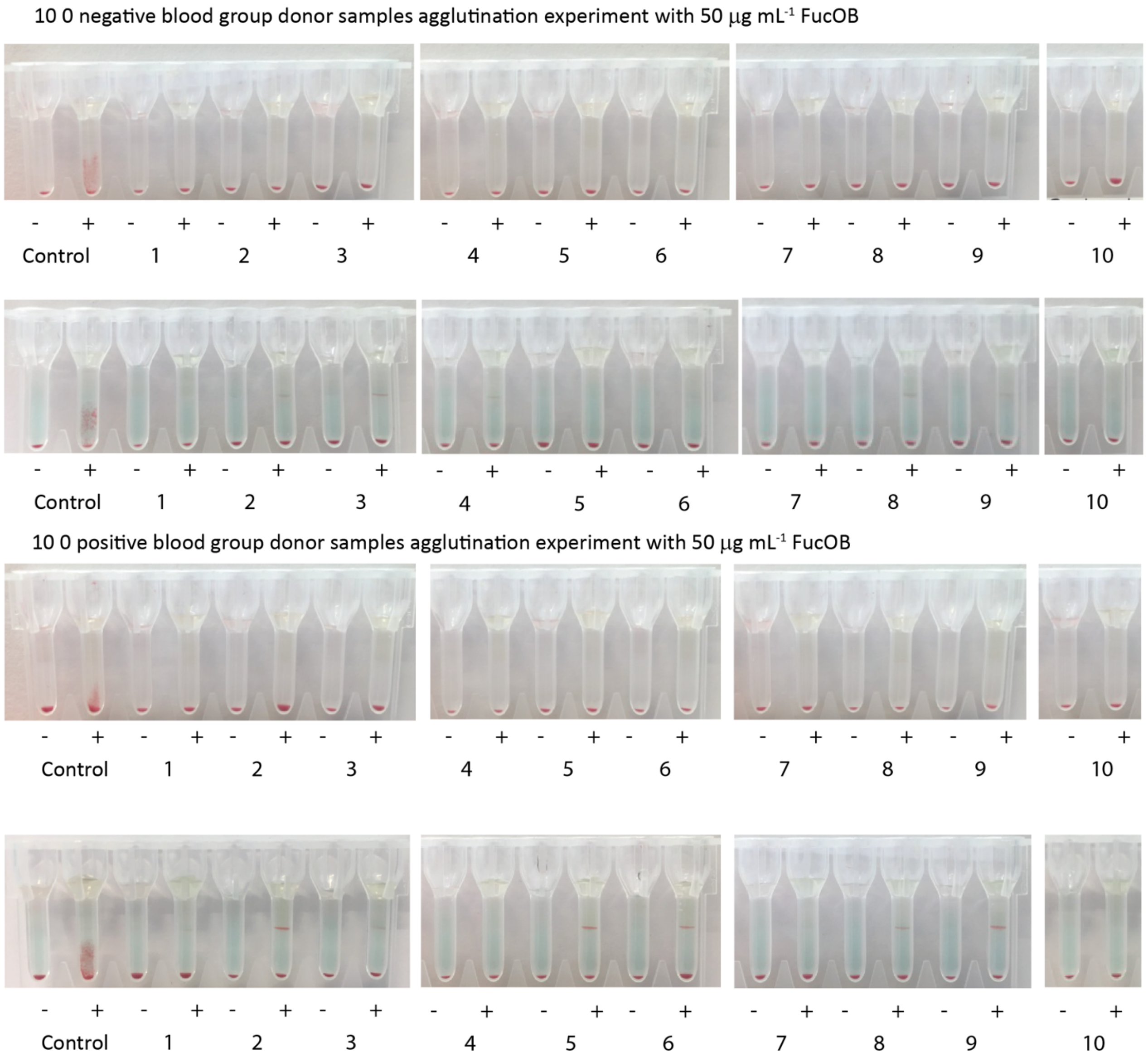

Supplementary Fig. 13 | FucOB converts both O^+ and O^- into rare Bombay type blood. Agglutination assay results from 10 different (represented from 1 to 10) O Rh negative blood group donor samples (in first and second rows) and 10 different (described from 1 to 10) O Rh positive blood group donor samples (in third and fourth rows). The agglutination assay was performed with enzymatically treated blood samples with FucOB at 50 μg mL^{-1} concentration and non-treated blood sample as a positive control. In the first and third rows the pictures of the assays performed in the gel column of DG Gel Neutral cards are shown. In the second and fourth rows the pictures of the assays performed in DG Gel Coombs cards are shown. Each sample was incubated in absence (-) and presence (+) of naturally containing anti-H antibodies Bombay serum. The pellet in the bottom of the gel column is a negative result, meaning that there is no agglutination or hemolysis in the sample. When clumps of cells appear throughout the gel column shows a positive result, meaning that cells are agglutinated in the sample. It is worth noting that each agglutination card has 8 buffered tubes to perform the experiments. Therefore, the results are shown in patches.

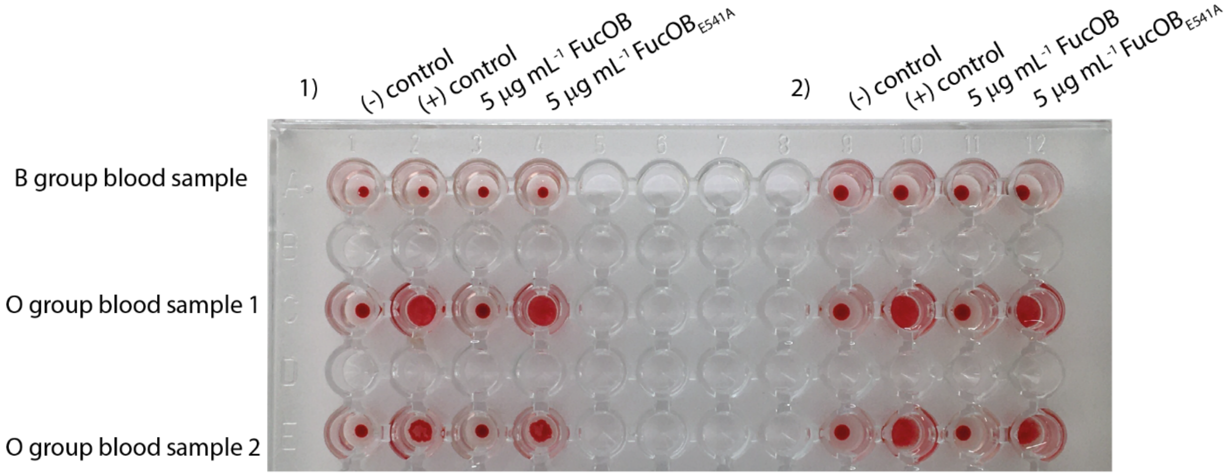

Supplementary Fig. 14 | FucOB conversion of universal O into rare Bombay type blood typing by anti-H lectin agglutination assay. Duplicate (experiment 1 on the left side and experiment 2 on the right side of the plate) agglutination assay experiments of three blood samples, (i) one B group blood sample as a negative control in the first row and (ii) two different O group blood samples (sample 1 in row 2 and sample 2 in row 3). In the first column, blood samples were incubated with PBS as a negative (-) control. In the second column, as a positive (+) control, blood samples were incubated with the anti-H lectin. In the third column, blood samples treated with 5 µg mL^{-1} of active wild type FucOB were incubated with the anti-H lectin. In the fourth column, blood samples treated with 5 µg mL^{-1} of the catalytically inactive $FucOB_{E541A}$ mutant were incubated with the anti-H lectin. The presence of RBCs pellet in the bottom indicates no agglutination (negative result) or hemolysis in the sample. A reddish RBC solution means cells agglutinated in the sample (positive result).

a

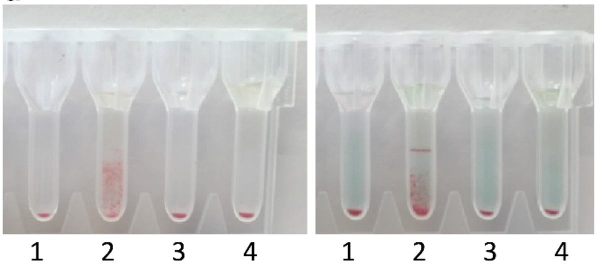

b

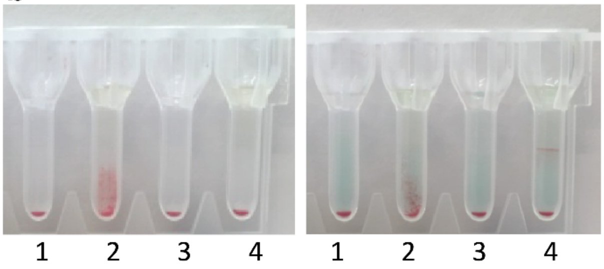

1- 0 μg mL^{-1} FucOB in fresh 0 blood sample
2- 0 μg mL^{-1} FucOB in fresh 0 blood sample +Bombay serum
3- 50 μg mL^{-1} FucOB in fresh 0 blood sample
4- 50 μg mL^{-1} FucOB in fresh 0 blood sample +Bombay serum

c

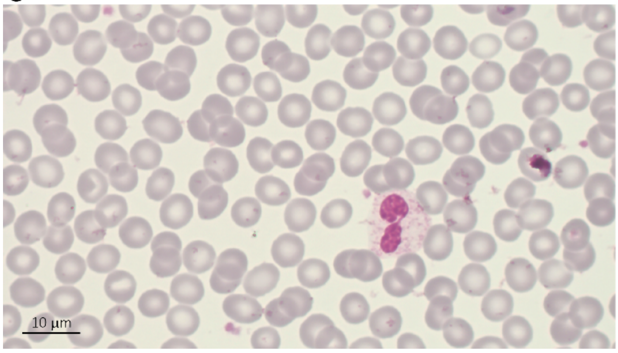

0 μg mL^{-1} FucOB in fresh 0 blood sample

d

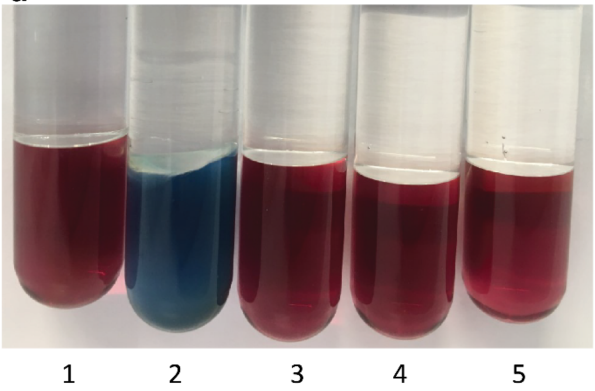

1- Positive control
2- Negative control
3-0 μg mL^{-1} FucOB in fresh 0 blood sample
4- 50 μg mL^{-1} FucOB in fresh 0 blood sample 1
5- 50 μg mL^{-1} FucOB in fresh 0 blood sample 2

Supplementary Fig. 15 | Viability and integrity of converted Bombay RBCs. Assays were performed in freshly drawn and non-washed O blood group samples. **a,b** Agglutination assay results from O blood group sample, directly treated with 0 μg ml^{-1} concentration active FucOB (1 and 2 gel columns) and with 50 μg ml^{-1} concentration active FucOB (3 and 4 gel-columns). In the left panel pictures of the assays performed in gel-column of DG Gel Neutral cards are shown. In the right panel pictures of the assays performed in DG Gel Coombs cards are shown. Each sample was incubated in absence (1 and 3 gel-columns) and presence (2 and 4 gel-columns) of naturally containing H antibodies Bombay serum. Pellet in the bottom of the gel-column is a negative result, meaning that there is no agglutination or hemolysis in the sample. When clumps of cells appear throughout the gel column shows a positive result, meaning that cells are agglutinated in the sample. Panel **a** shows the results from donor 1 and panel **b** from donor 2. It is worth noting that each agglutination card has 8 buffered tubes to perform the experiments. Therefore, the results are shown in patches. **c** Blood sample smear picture after an incubation at 37ºC without enzyme. RCBs are shown with normal biconcave morphology in both analyzed duplicates. **d** G6DP activity colorimetric assay results. The red color in the tube solution means that G6DP present in RBC is active, and the blue color indicates that it has lost its activity. 1 and 2 tubes are positive and negative controls, respectively. Tube 3 shows a blood sample after incubation at 37ºC without FucOB. Tube 4 and 5 show two different blood samples after an incubation at 37ºC in presence of 50 μg mL^{-1} of active FucOB.

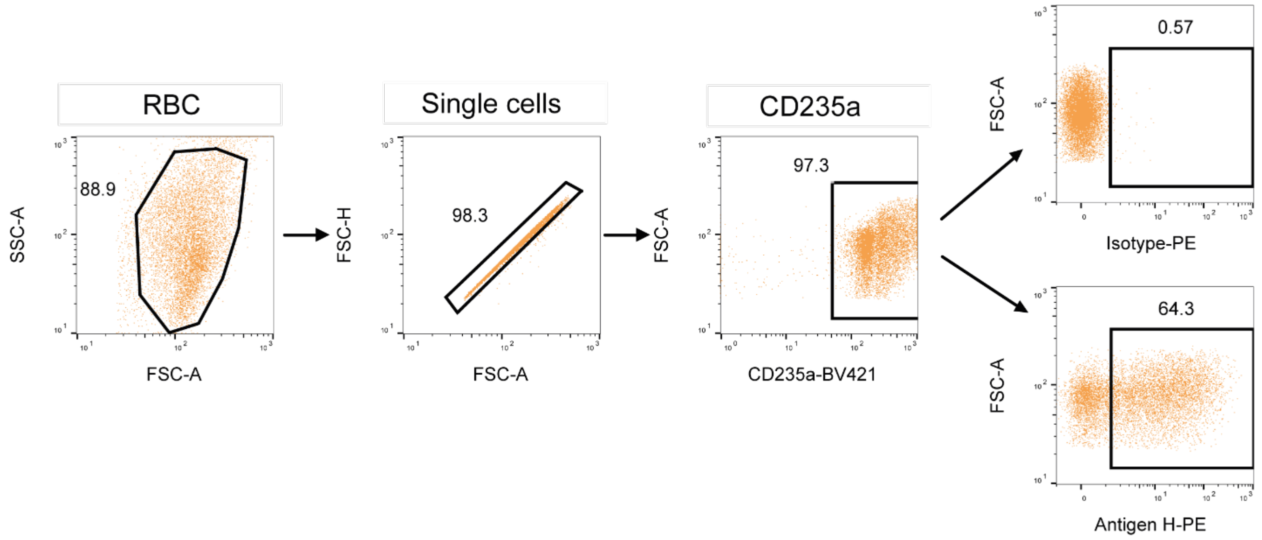

Supplementary Fig. 16 | Gating strategy for the analysis of antigen H expression in RBCs. Dot plot graphs showing the gating strategy used for the analysis of antigen H expression on O type RBCs. Data from a representative example is shown. RBCs were electronically gated based on their forward (FSC) and side scatter (SSC) parameters and then single cells were selected. Next, the population positive for the CD235a (Glycophorin A), a transmembrane glycoprotein expressed by erythrocytes, was selected. Finally, the frequency of cells positive for antigen H were analyzed. The determination of the population positive for antigen H was based in the isotype control.

3. Supplementary References

1. Vieira, P. S. *et al.* Xyloglucan processing machinery in Xanthomonas pathogens and its role in the transcriptional activation of virulence factors. *Nat Commun* **12**, 4049 (2021).
2. Katayama, T. *et al.* Molecular cloning and characterization of Bifidobacterium bifidum 1,2-α-L-fucosidase (AfcA), a novel inverting glycosidase (glycoside hydrolase family 95). *J Bacteriol* **186**, 4885–4893 (2004).
3. Nagae, M. *et al.* Structural basis of the catalytic reaction mechanism of novel 1,2-α-L-fucosidase from Bifidobacterium bifidum. *Journal of Biological Chemistry* **282**, 18497–18509 (2007).
4. Déjean, G., Tauzin, A. S., Bennett, S. W., Creagh, A. L. & Brumer, H. Adaptation of syntenic xyloglucan utilization loci of human gut Bacteroidetes to polysaccharide side chain diversity. *Appl Environ Microbiol* **85**, e01491-19 (2019).
5. Sela, D. A. *et al.* Bifidobacterium longum subsp. infantis ATCC 15697 α-fucosidases are active on fucosylated human milk oligosaccharides. *Appl Environ Microbiol* **78**, 795–803 (2012).
6. Larsbrink, J. *et al.* A complex gene locus enables xyloglucan utilization in the model saprophyte Cellvibrio japonicus. *Mol Microbiol* **94**, (2014).
7. Fan, S. *et al.* Cloning, characterization, and production of three α-l-fucosidases from Clostridium perfringens ATCC 13124. *J Basic Microbiol* **56**, (2016).
8. Pichler, M. J. *et al.* Butyrate producing colonic Clostridiales metabolise human milk oligosaccharides and cross feed on mucin via conserved pathways. *Nat Commun* **11**, 3285 (2020).
9. Wu, H. *et al.* Fucosidases from the human gut symbiont Ruminococcus gnavus. *Cellular and Molecular Life Sciences* **78**, 675–693 (2021).
10. Hobbs, J. K., Pluvinage, B., Robb, M., Smith, S. P. & Boraston, A. B. Two complementary α-fucosidases from Streptococcus pneumoniae promote complete degradation of host-derived carbohydrate antigens. *Journal of Biological Chemistry* **294**, (2019).
11. Tiansheng Li, Juan Ye, Lei Wang, Lin Zou, Yameng Guo, Linlin Hou, Danfeng Shen, Xiaohong Cai, Haobo Huang, Guiqin Sun, L. C. Bacterial fucosidase enables the production of Bombay red blood cells. *bioRxiv* (2019).
12. Rogowski, A. *et al.* Glycan complexity dictates microbial resource allocation in the large intestine. *Nat Commun* **6**, (2015).
13. Ndeh, D. *et al.* Complex pectin metabolism by gut bacteria reveals novel catalytic functions. *Nature* **544**, (2017).
14. Günl, M. *et al.* AXY8 encodes an α-fucosidase, underscoring the importance of apoplastic metabolism on the fine structure of Arabidopsis cell wall polysaccharides. *Plant Cell* **23**, (2011).
15. Bauer, S., Vasu, P., Persson, S., Mort, A. J. & Somerville, C. R. Development and application of a suite of polysaccharide-degrading enzymes for analyzing plant cell walls. *Proc Natl Acad Sci U S A* **103**, (2006).
16. Ishimizu, T., Hashimoto, C., Takeda, R., Fujii, K. & Hase, S. A novel α1,2-L-fucosidase acting on xyloglucan oligosaccharides is associated with endo-β-mannosidase. *J Biochem* **142**, (2007).
