# Supplementary material for: Turning universal O into rare Bombay type blood
Source: Nat Commun. 2023 Mar 30;14:1765. doi: 10.1038/s41467-023-37324-z (PMC10063614; doi:10.1038/s41467-023-37324-z)
Supplement: Supplementary file 2 — Reporting Summary [file 41467_2023_37324_MOESM2_ESM.pdf]

nature portfolio

Corresponding author(s): Beatriz Trastoy
Jonathan Sjögren
Marcelo E. Guerin

Last updated by author(s): Mar 1, 2023

Reporting Summary

Nature Portfolio wishes to improve the reproducibility of the work that we publish. This form provides structure for consistency and transparency in reporting. For further information on Nature Portfolio policies, see our Editorial Policies and the Editorial Policy Checklist.

Statistics

For all statistical analyses, confirm that the following items are present in the figure legend, table legend, main text, or Methods section.

n/a	Confirmed	
☐	☒	The exact sample size (n) for each experimental group/condition, given as a discrete number and unit of measurement
☐	☒	A statement on whether measurements were taken from distinct samples or whether the same sample was measured repeatedly
☒	☐	The statistical test(s) used AND whether they are one- or two-sided<br>*Only common tests should be described solely by name; describe more complex techniques in the Methods section.*
☒	☐	A description of all covariates tested
☒	☐	A description of any assumptions or corrections, such as tests of normality and adjustment for multiple comparisons
☐	☒	A full description of the statistical parameters including central tendency (e.g. means) or other basic estimates (e.g. regression coefficient) AND variation (e.g. standard deviation) or associated estimates of uncertainty (e.g. confidence intervals)
☒	☐	For null hypothesis testing, the test statistic (e.g. F, t, r) with confidence intervals, effect sizes, degrees of freedom and P value noted<br>*Give P values as exact values whenever suitable.*
☒	☐	For Bayesian analysis, information on the choice of priors and Markov chain Monte Carlo settings
☒	☐	For hierarchical and complex designs, identification of the appropriate level for tests and full reporting of outcomes
☒	☐	Estimates of effect sizes (e.g. Cohen's d, Pearson's r), indicating how they were calculated

Our web collection on statistics for biologists contains articles on many of the points above.

Software and code

Policy information about availability of computer code

Data collection	Reverse phase HPLC on a Vanquish Duo UHPLC system equipped with a MSPac DS-10 desalting cartridge (both Thermo Fisher)<br>AMBER 20 package<br>MACSQuant Analyzer

Data analysis	Vanquish Duo UHPLC system<br>xds<br>Ccp4 8.0<br>Phenix 1.20.1<br>WinCoot 0.9.8.1<br>Chimera 1.16<br>HOLLOW<br>DALI<br>PISA<br>Multiple Align Show server<br>GLYCAM<br>AutoDock Vina 1.5.7<br>AMBER 20 package<br>FlowJoTM v10.4

For manuscripts utilizing custom algorithms or software that are central to the research but not yet described in published literature, software must be made available to editors and reviewers. We strongly encourage code deposition in a community repository (e.g. GitHub). See the Nature Portfolio guidelines for submitting code & software for further information.

Data

Policy information about availability of data

All manuscripts must include a data availability statement. This statement should provide the following information, where applicable:

- Accession codes, unique identifiers, or web links for publicly available datasets
- A description of any restrictions on data availability
- For clinical datasets or third party data, please ensure that the statement adheres to our policy

The atomic coordinates and structure factors have been deposited with the Portein Data Bank, access codes 7ZNZ (https://www.rcsb.org/structure/unreleased/7ZNZ) (FucOBWT) and 7ZO0 (https://www.rcsb.org/structure/unreleased/7ZO0) (FucOBE541A). Previously published PDB structures used in this study are available under the accession codes: 2EAB[https://www.rcsb.org/structure/2EAB], 7KMQ [https://www.rcsb.org/structure/7KMQ], , 2RDY [https://www.rcsb.org/structure/2RDY], 2EAD[https://www.rcsb.org/structure/2EAD], 2EAE [https://www.rcsb.org/structure/2EAE], 2EAC [https://www.rcsb.org/structure/2EAC] and 4UFC [https://www.rcsb.org/structure/4UFC].Other data are available from the corresponding authors under reasonable request. Source data are provided with this paper.

Human research participants

Policy information about studies involving human research participants and Sex and Gender in Research.

Reporting on sex and gender	This is not relevant for this study and therefore this information has not been collected. The blood samples were supplied from anonymous healthy donors blood samples from the Blood Bank of Cruces Hospital regardless of sex and gender.
Population characteristics	12 0 positive and 10 0 negative blood group samples<br>1 B positive blood group sample<br>1 Bombay blood group sample<br>All of the samples were collected from anonymous healthy donors blood from Blood Bank
Recruitment	anonymous healthy donors blood samples from the Blood Bank of Cruces Hospital
Ethics oversight	Ethics Committee for Clinical Research of Cruces University Hospital (CEI E21/65)<br>IM.CTCV-HEM-01A.02

Note that full information on the approval of the study protocol must also be provided in the manuscript.

Field-specific reporting

Please select the one below that is the best fit for your research. If you are not sure, read the appropriate sections before making your selection.

☒ Life sciences ☐ Behavioural & social sciences ☐ Ecological, evolutionary & environmental sciences

For a reference copy of the document with all sections, see nature.com/documents/nr-reporting-summary-flat.pdf

Life sciences study design

All studies must disclose on these points even when the disclosure is negative.

Sample size	As it is described in methods section, we performed the experiments in duplicates or triplicates, with the exception of O blood group enzymatic conversion assay which was performed in 20 O group blood samples (10 Rh positive and 10 Rh negative sanples), in order to

perform the experiment in larger number of samples. We have included this information in the relevant figure legends.

Data exclusions: No data was excluded

Replication:
Triplicate experiments were performed in activity assays.
Duplicate experiments were performed in Anti-H lectin agglutination assays.
Duplicate experiments were performed in Glucose 6 Phosphate Dehydrogenase Assays.
The experiment was repeated 10 times in DG Gel column agglutination assays.
Duplicate experiments were performed in FACs assays.
All the experiments were reproducible.

Randomization: This is not relevant for this study because we did not need to prevent bias in our experiments

Blinding: This is not relevant for this study because we did not need to prevent bias in our experiments

Reporting for specific materials, systems and methods

We require information from authors about some types of materials, experimental systems and methods used in many studies. Here, indicate whether each material, system or method listed is relevant to your study. If you are not sure if a list item applies to your research, read the appropriate section before selecting a response.

Materials & experimental systems

n/a	Involved in the study
☐	☒ Antibodies
☒	☐ Eukaryotic cell lines
☒	☐ Palaeontology and archaeology
☒	☐ Animals and other organisms
☒	☐ Clinical data
☒	☐ Dual use research of concern

Methods

n/a	Involved in the study
☒	☐ ChIP-seq
☐	☒ Flow cytometry
☒	☐ MRI-based neuroimaging

Antibodies

Antibodies used:
Anti-blood group H ab antigen antibody from abcam [97-I] (ab24213)
Anti-blood Group H n/ab antigen antibody [86-M] (AGM-022YJ) from Creative Biolabs
mouse IgM [B11/7]-Isotype control from abcam (ab91545)
PE goat anti-mouse IgM (Heavy chain) cross-adsorbed secondary antibody from Thermo Fisher Scientific (M31504)
BV421 Mouse Anti-Human CD235a from Biosciences (cat. number 562938)

Validation:
Anti-blood group H antigen antibody validation and specification can be found at manufacturer's webpage (https://www.abcam.com/blood-group-h-ab-antigen-antibody-97-i-ab24213.html) and and data is provided in the manuscript.
Anti-blood Group H n/ab antigen antibody [86-M] (AGM-022YJ) from Creative Biolabs validation and specification can be found at manufacturer's webpage (https://www.creative-biolabs.com/anti-glycan-antibodies/anti-blood-group-h-n-ab-antigen-antibody-86-m-22.htm) and data is provided in the manuscript.
mouse IgM [B11/7]-Isotype control from abcam (ab91545) validation and specificities can be found at manufacturer's webpage (https://www.abcam.com/mouse-igm-b117-isotype-control-ab91545.html) and data is provided in the manuscript.
BV421 Mouse Anti-Human CD235A validation and specificities can be found at manufacturer's webpage (https://www.bdbiosciences.com/en-us/products/reagents/flow-cytometry-reagents/research-reagents/single-color-antibodies-ruo/bv421-mouse-anti-human-cd235a.562938) and the corresponding data is provided in the manuscript.

Flow Cytometry

Plots

Confirm that:

☒ The axis labels state the marker and fluorochrome used (e.g. CD4-FITC).

☒ The axis scales are clearly visible. Include numbers along axes only for bottom left plot of group (a 'group' is an analysis of identical markers).

☒ All plots are contour plots with outliers or pseudocolor plots.

☒ A numerical value for number of cells or percentage (with statistics) is provided.

Methodology

Sample preparation	Enzymatically treated O type RBCs were diluted (1:10) in PBS and then 1µl of diluted blood was added to 100µL of staining buffer (PBS + 1% fetal bovine serum). Next, cells were incubated with (1:100) mouse anti-blood group H ab antigen antibody (97-I), (1:10) mouse anti-blood group H n/ab antigen antibody (86-M) (from Creative Biolabs) or with (1:10) mouse IgM Isotype control (B11/7) or 30 minutes on ice. Then, cells were washed twice with staining buffer and incubated with (1:100) PE goat anti-mouse IgM antibodyor 30 minutes at RT. Then, cells were washed twice with staining buffer and incubated with BV421 mouse anti-CD235a (GA-R2) from BD Bioscience for 30 minutes on ice. Lastly, samples were washed once and resuspended in staining buffer.
Instrument	MACSQuant Analyzer 10 flow cytometer (Miltenyi Biotec)
Software	FlowJoTM v10.4.
Cell population abundance	The population positive for the CD235a in a representative O blood group sample is 97.3%<br>The frequency of cells positive for antigen H (abcam; 97-I) in a representative O blood group sample is 64.2%<br>The frequency of cells positive for antigen H (creative biolabs; 86-M) in a representative O blood group sample is 56.7%
Gating strategy	RBCs were electronically gated based on their forward (FSC,) and side scatter (SSC) parameters (82.6% population) and then single cells were selected (98.3% population). Next, the population positive for the CD235a (Glycophorin A), a transmembrane glycoprotein expressed by erythrocytes, was selected (97.3% of population). Finally, the frequency of cells positive for antigen H were analyzed (64.3% of population with abcam 97-I antibody and 56.7% with creative biolabs 86-M antibody). The determination of the population positive for antigen H was based in the isotype control.

☒ Tick this box to confirm that a figure exemplifying the gating strategy is provided in the Supplementary Information.
